# CRISPR/Cas9-based iterative multi-copy integration for improved metabolite yields in *Saccharomyces cerevisiae*

**DOI:** 10.1016/j.synbio.2025.02.016

**Published:** 2025-03-05

**Authors:** Ximei Chen, Chenyang Li, Xin Qiu, Ming Chen, Yongping Xu, Shuying Li, Qian Li, Liang Wang

**Affiliations:** a School of Biological Engineering, Dalian Polytechnic University, Dalian, 116034, China; b School of Life and Health, Dalian University, Dalian, 116622, China; c Postdoctoral Workstation of Dalian SEM Bio-Engineering Technology Co. Ltd., Dalian, 116000, China; d School of Bioengineering, Dalian University of Technology, Dalian, 116024, China

**Keywords:** Multi-copy integration, *Saccharomyces cerevisiae*, CRISPR/Cas9, Ergothioneine, Cordycepin

## Abstract

High-copy integration of key genes offers a promising strategy for efficient biosynthesis of valuable natural products in *Saccharomyces cerevisiae*. However, traditional multi-copy gene integration methods meet challenges including low efficiency and labor-intensive screening processes. In this study, we developed the IMIGE (Iterative Multi-copy Integration by Gene Editing) system, a CRISPR/Cas9-based approach that exploits both δ and rDNA repetitive sequences for simultaneous multi-copy integrations in *S. cerevisiae*. This system combines the mixture of Cas9-sgRNA expression vectors with a split-marker strategy for efficient donor DNA assembly in vivo and enables rapid, iterative screening through growth-related phenotypes. When applied to the biosynthesis of ergothioneine and cordycepin, the IMIGE system achieved significant yield improvements, with titers of 105.31 ± 1.53 mg/L and 62.01 ± 2.4 mg/L, respectively, within just two screening cycles (5.5–6 days in total). These yields represent increases of 407.39 % and 222.13 %, respectively, compared to the strains with episomal expression. By streamlining the integration process, utilizing growth-based selection, and minimizing screening demands in both equipment and labor, the IMIGE system could provide an efficient and scalable platform for high-throughput strain engineering, facilitating enhanced microbial production of a wide range of bioproducts.

## Graphical abstract

**Figure:**
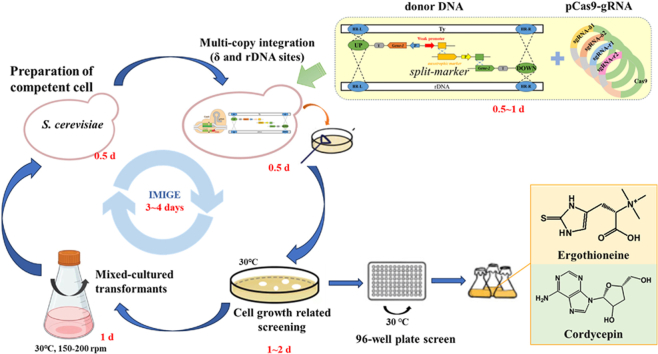

## 1 Introduction

Small-molecule natural products derived from plants, animals, and microbes play vital roles in medicine due to their antioxidant, antibacterial, anti-inflammatory, and anticancer properties, with approximately 40 % of modern therapeutic drugs originating from these sources [1]. However, traditional production methods, such as plant extraction and chemical synthesis, face significant challenges, including slow growth rates, high resource demands, low yields, and high costs. Modern biotechnologies covering synthetic biology, metabolic engineering, and gene-editing can offer more efficient, cost-effective, and sustainable alternatives for natural product synthesis, addressing the limitations of conventional methods.

The yeast *Saccharomyces cerevisiae* is widely used in large-scale fermentation due to its safety, ease of cultivation, well-characterized genetics, and compatibility with advanced gene-editing technologies [2]. Its ability to express cytochrome P450 enzymes makes it particularly suited for heterologous biosynthesis, especially for plant-derived natural products. Various research groups have leveraged *S. cerevisiae* to synthesize compounds such as artemisinic acid [3], cannabinoids [4], vinblastine [5], jasmonates [6], and the vaccine adjuvant QS-21 [7].

Metabolic engineering strategies including overexpression of heterologous genes are commonly applied to optimize biosynthetic pathways in yeast for efficient natural product synthesis [8,9]. However, these efforts often have to face challenges such as poor gene expression leading to low yields. Traditional expression systems may yield inconsistent results due to variations in strain genetic backgrounds and product characteristics. Due to the highly efficient homologous recombination, *S. cerevisiae* is well-suited for stable gene integration, though single-copy integration often doesn't result in best yields [10]. Increasing gene copy numbers can enhance the transcription level, protein expression, and product yields. Thus, multi-copy gene integration, which commonly uses repeated sequences such as δ or rDNA sites, has emerged as a central strategy in yeast metabolic engineering. The δ sequence, with over 300 copies in the yeast genome [11], and the rDNA sequence, with approximately 200 copies [12], both support high-efficiency integration. Shi et al. further demonstrated the potential of this approach by integrating up to 18 copies of a gene using Di-CRISPR, resulting in significantly improved product yields [13].

Despite its potential, multi-copy site integration also faces limitations, such as its restricted target gene copies, long operational cycles, and reduced efficiency especially when larger DNA fragments are involved. Especially, relying solely on rDNA or δ sites underutilizes the full potential of these sequences, while traditional screening processes often involve multiple verification steps (PCR, sequencing, fermentation, and testing of product content), resulting in workflows that are not only time-consuming and complex but also heavily dependent on specialized equipment.

In this study, we developed a novel approach that combines nutritional deficiency complementation with δ sequence/rDNA site integration and CRISPR-Cas9 gene editing technology to enable the rapid obtainment of *S. cerevisiae* recombinants with high-copy integrations of large DNA fragments. Using this method, we successfully generated *S. cerevisiae* strains capable of producing cordycepin and ergothioneine.

## 2 Materials and methods

### 2.1 Strains and media

All strains used in this study are shown in Table S1. *Escherichia coli* DH5α was used for plasmid synthesis or subcloning and cultured at 37 °C in LB medium (10 g/L tryptone, 5 g/L yeast extract, 10 g/L NaCl), with 100 mg/L ampicillin for plasmid selection if needed. *S. cerevisiae* BY4741 was used as the host for gene insertion and cultured at 30 °C in either YPD or YNB medium (without amino acids/ammonium sulfate), supplemented with amino acids as needed. YNB and YPD media were sourced from YuanYe (Shanghai, China) and MeilunBio (Dalian, China), respectively. Ampicillin was purchased from BBI (Shanghai, China).

### 2.2 DNA manipulation, plasmid construction and yeast transformation

The plasmids used in this study are listed in Table S1. The pECAS9-KlURA3-gRNA plasmid was used for Cas9 and gRNA expression. Gibson assembly was performed using the ClonExpress Ultra One-Step Cloning Kit (Vazyme, Nanjing, China). All PCR primers are detailed in Table S2. Briefly, targeted fragments were amplified using primers with homologous arms with PrimeSTAR® Max DNA Polymerase (Takara, Beijing, China) and assembled using Gibson assembly. The CHOPCHOP website (http://chopchop.cbu.uib.no/) was used to design sgRNAs (Table S3), and the Cas9 system was utilized for single-site gene insertions. Overlap PCR was employed to assemble multiple gene fragments.

For yeast transformation, the key genes *EGT1&2* (for ergothioneine biosynthesis) [14] and *CNS1&2* (for cordycepin biosynthesis) [15] were integrated into the *S. cerevisiae* genome. Fragments (delta1, delta2, rDNA1, rDNA2) were amplified from the yeast genome using F/R201-204 primers, and gene expression cassettes (P301–P304 and Pg201-Pg215 for *EGT1&2*, and P321–P326 for *CNS1&2*) were amplified from the plasmids in Table S1. The *MET17* selection marker was amplified from pYES2-URA3-MET17 using F213/R213. These PCR products were purified from agarose and assembled using overlap PCR. Yeast was routinely transformed by electroporation method, with ∼2 μg of DNA per transformation.

### 2.3 Iterative screening of the engineered yeast

**Fig. 1:**
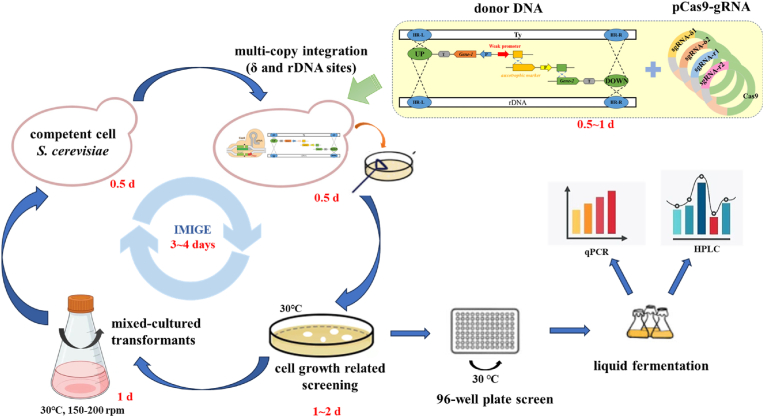
Schematic diagram for the construction and operation of IMIGE system. The IMIGE system comprises three steps: constructing Cas9-sgRNA expression vectors, preparing linear donor DNA fragments, and iterative yeast transformation and screening. Four pCas9-sgRNA plasmids are designed to co-express Cas9 and sgRNAs, targeting δ elements and the rDNA region to enable simultaneous integration at both sites. Donor DNA fragments are prepared for assembly in vivo using a split-marker strategy, consisting of homologous arms, target gene expression cassettes, and a selectable marker (such as *MET17*) driven by a weak promoter to facilitate high-copy integration. Co-transformation of donor DNA with the mixture of Cas9-sgRNA vectors into the yeast strain BY4741 is followed by screening on selective medium. Transformants with high-copy integrations are enriched through mixed-culture growth in drop-out liquid medium, for only transformants with abundant expression of the selectable marker allow robust growth in selective medium. Iterative transformations progressively increase integration copy numbers of target genes, streamlining the process by reducing the need for colony picking and verification.

The screening of engineered yeast strains involved iterative yeast transformation and selection rounds to obtain optimal transformants (Fig. 1). The number of iterations was correlated with desired gene copies. Following each round of yeast electroporation, transformants were plated on auxotrophic solid media for a specific amino acid, and incubated at 30 °C. Once 5–10 transformants appeared on the plates, approximately 5 mL of sterile saline was added to each plate, and all colonies were scraped and overnight cultured. This culture then served as the host strain for the next round of transformation. Positive clones obtained through iterative screening were inoculated into 96-well plates, adding 1 mL of drop-out liquid medium in accordance with the solid media mentioned above to each well. The plates were then incubated statically at 30 °C. The strains with a higher number of cells at the bottom of each well were considered to have a higher copy number in targeted gene cassettes.

### 2.4 Liquid fermentation

After screening, the engineered yeast was cultured for ergothioneine or cordycepin production. Precultures were initiated by inoculating a single colony into 100 mL synthetic complete (SC) medium at 30 g/L glucose (Table S6), then incubated at 30 °C, 250 rpm for 24 h. Afterwards culturing in 250 mL flasks containing 50 mL of medium was performed, with an initial OD_600_ of 0.2.

### 2.5 Induction of gene expression using galactose-inducible promoters

The galactose-inducible promoters *P*_*GAL1*_ and *P*_*GAL10*_ were used in *S. cerevisiae* BY4741 to drive the expression of genes for cordycepin or ergothioneine production. A single yeast colony was inoculated into SC medium with 20 g/L glucose and incubated overnight at 30 °C with shaking. The culture was maintained under the same conditions until reaching the mid-log phase (OD_600_ = 0.6–0.8). Cells were then harvested by centrifugation at 3000×*g*, resuspended in SC medium with 20 g/L galactose (initial OD_600_ = 0.2), and fermented for 144 h at 30 °C with shaking at 200 rpm.

### 2.6 Copy number and passage stability analysis by qPCR

Yeast cells were grown overnight in YPD medium, then transferred to fresh YPD and cultured to an OD_600_ of 0.6–0.8. Genomic DNA was extracted using the SDS method [16]. Primers for qPCR are listed in Table S2. Gene expression was quantified using a qPCR kit (TransStart® Top Green qPCR Super Mix) with *ACT1* as the reference gene. Quantification of gene expression was performed with the 2^−ΔΔCt^ method.

To evaluate the genetic stability of genomic integration, we assessed the passage stability of the engineered strains. The strains were cultured in SC medium (without nutrient deficiencies) and passaged every 12 h for a total of 48 h. Cells were collected, genomic DNA was extracted, and the copy number of the integrated target gene (excluding the episomal expression strain) was quantified by qPCR.

### 2.7 Metabolite analysis

To determine extracellular content of ergothioneine or cordycepin, 2 mL of fermentation broth was centrifuged at 3000×*g* for 5 min, and the supernatant was analyzed by a Waters HPLC system with UV detection. Ergothioneine was detected at 254 nm using the Xcharge C18 column (4.6 × 250 mm, 5 μm, Acchrom, China) with 0.1 % formic acid as the mobile phase, The column temperature was maintained at 25 °C, with a flow rate of 0.5 mL/min from 0 to 20 min, increasing to 0.8 mL/min from 21 to 29 min, and then returning to 0.5 mL/min from 30 to 35 min. The injection volume was 10 μL. Cordycepin was detected at 260 nm by the Venusil MP C18 column (4.6 × 250 mm, 5 μm, Agela) with 20 % methanol as the mobile phase. The column temperature was maintained at 25 °C, and the flow rate was set at 0.8 mL/min. The injection volume was 10 μL.

## 3 Results and discussion

### 3.1 Development of IMIGE system

The high-level expression of key genes is essential for efficient and stable metabolite accumulation, and multi-copy genome integration provides an effective strategy to achieve this goal [11,17]. In *Saccharomyces cerevisiae*, the repeated δ or rDNA genomic sites offer ideal loci for multi-copy integration of heterologous genes. Although these sites have each been widely used individually in yeast strain engineering, no reports have yet explored the simultaneous utilization of both δ and rDNA sites for multi-copy integration to the best of our knowledge. Targeting both sites concurrently has the potential to increase the chromosomal integration copies of the target gene with greater ease, which motivated us to investigate the use of both δ and rDNA sites for the multi-copy integration of exogenous genes. Traditional yeast multi-copy integration studies typically require verification of transformants, such as through PCR or HPLC, following yeast transformation to identify optimal strains. These procedures are time-consuming and heavily dependent on detection instruments.

The advent of CRISPR-Cas9 gene editing has revolutionized multi-gene modifications, offering significant advantages over traditional methods, including in *S. cerevisiae*. To enable rapid generation of yeast strains with high gene copy numbers, we developed the IMIGE system, an iterative multi-copy integration system based on CRISPR/Cas9 gene editing (Fig. 1). This system maximizes the use of δ and rDNA sites to increase the number of integration targets for the target gene more easily, and simultaneously greatly shortens the time required to construct high-copy strains through a rapid screening strategy focused only on cell growth-related phenotypes. As Fig. 1 showed, the IMIGE system comprises three key components: (i) constructing the Cas9-sgRNA expression vector; (ii) preparing linear donor DNA fragments; (iii) iterative yeast transformation and screening.

To achieve efficient gene editing in yeast, it is critical to optimize the expression of both Cas9 and sgRNA. The gene-editing plasmid pCas9-sgRNA is specifically engineered for the co-expression of Cas9 and sgRNAs targeting designated genomic loci in yeast, such as δ elements or the rDNA region, enabling precise genomic modifications. Cas9 and sgRNAs are expressed under yeast-compatible promoters, enhancing editing efficiency. To reduce cytotoxicity associated with prolonged Cas9 activity, both Cas9 and the sgRNA scaffold are episomally expressed with *URA3* as the selectable marker, allowing for transient expression during the editing process. The use of plasmid also accommodates flexible sgRNA insertion through seamless cloning like Gibson assembly, facilitating efficient multi-loci targeting. To further enhance editing efficiency, two pairs of sgRNAs are designed to target δ elements or the rDNA region each, resulting in four distinct Cas9-sgRNA expression vectors (Table S1). During each transformation, all four plasmids are introduced simultaneously, ensuring comprehensive targeting and modification across selected loci.

The second phase of this process involves preparing donor DNA fragments, which contain several essential components: homologous arms, a target gene expression cassette, and a selection marker. These elements can be assembled in vitro into a single fragment, often exceeding 10 kb in length, making the ligation process both complex and time-consuming. To address this challenge, the system leverages extremely high capability of homologous recombination in *S. cerevisiae* with a split-marker strategy (Fig. 1), where the donor DNA is divided into shorter fragments, each under 5000 bp. This approach enables yeast cells to assemble and integrate the fragments *via* homologous recombination in vivo, significantly streamlining the assembly process and saving time. The auxotrophic *S. cerevisiae* strain BY4741, deficient in uracil, histidine, methionine, and leucine, serves as the host cell. A nutrient-related deficiency gene, such as *MET17* (also known as *MET15*), is employed as the selectable marker. To facilitate high-copy integration during iterative transformations, weak promoters (e.g., *P*_*BTS1*_, *P*_*PDA1*_, *P*_*ERG1*_) [18,19] are chosen to drive the expression of the selected nutrient deficiency gene, and only strains carrying high-copy integration of the donor fragments allow robust growth on drop-out medium, which could compensate for the insufficient expression of the nutrient-deficient gene caused by the weak promoter.

The third step involves introducing the recombinant vector pCas9-sgRNA and donor DNA into yeast cells *via* electroporation or chemical transformation, followed by iterative selection to screen for the desired strain considering the limited increase in gene copies within each round. Yeast cells are plated on drop-out media immediately after transformation. Transformants with high-copy integrations of the donor DNA, which ensures the high copy of the growth-related selection marker simultaneously, would proliferate much more rapidly on selective plates. Once 5–10 colonies appear, all transformants are collected and further cultured in liquid drop-out medium, able to outcompete others due to their enhanced specific growth rate, gradually emerging as the dominant population. The enriched culture is then used to prepare competent cells for the next round of transformation, thereby incrementally increasing gene integration copy numbers round by round. This approach of mixed-culture enrichment and iterative transformation minimizes labor needed, such as colony picking and PCR verification, and ensuring that high-copy integrant strains are carried forward in subsequent rounds, preserving promising strains with minimal validation.

Compared to traditional methods for constructing multi-copy yeast strains, the IMIGE system optimizes several critical stages, including integration site selection, donor DNA fragment construction, transformant screening. Using this system, multi-copy yeast strains can be easily constructed within 3–4 days per screening cycle, saving 1–3 days compared to conventional approaches.

### 3.2 Construction of engineered yeast for ergothioneine production based on the IMIGE system

Ergothioneine, a histidine-derived thiol found in organisms such as mushrooms, cyanobacteria, and mycobacteria, is known for its potent antioxidant properties. It plays a key role in cellular protection, detoxification, DNA preservation, and immune function. Due to its health benefits, ergothioneine is now used in various industries, including cosmetics [20], functional foods [21], and biomedicine [22]. It was approved as a novel food ingredient by the European Commission in 2017 and granted GRAS (Generally Recognized as Safe) status by the US FDA in 2019.

**Fig. 2:**
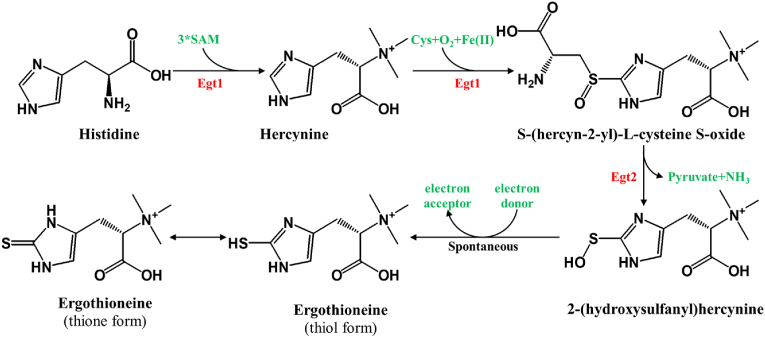
The biosynthetic pathway of ergothioneine in eukaryotes. Ergothioneine biosynthesis in eukaryotes involves two key enzymes, Egt1 and Egt2. Egt1, with methyltransferase and 5-histidylcysteine sulfoxide synthase activities, catalyzes the conversion of deacetylcysteine to its sulfoxide and methylates either cysteine or histidine, resulting the formation of deacetylcysteine. Egt2, a hercynylcysteine S-oxide lyase, completes the process by forming ergothioneine after C–S bond formation.

Traditionally, ergothioneine has been obtained through costly and low-yield methods, such as chemical synthesis and natural extraction [22]. However, microbial biosynthesis offers significant advantages, including lower costs, higher yields, and greater environmental sustainability, making it a promising approach for large-scale production. The biosynthetic pathway for ergothioneine, present in organisms like *Mycobacterium* species and *Schizosaccharomyces pombe* [23], is driven in eukaryotes by two key enzymes (Fig. 2): Egt1, which converts deacetylcysteine to its sulfoxide and methylates cysteine or histidine, and Egt2, which completes ergothioneine synthesis by forming a C–S bond with the sulfoxide [24].

**Fig. 3:**
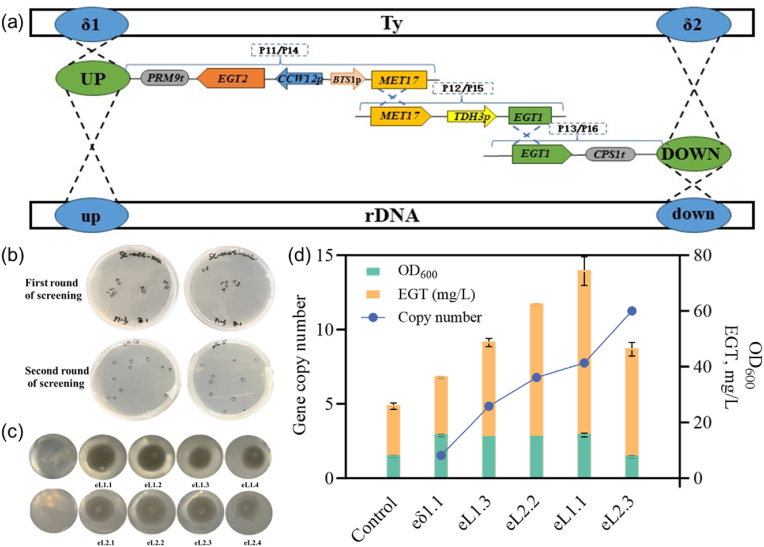
Application of IMIGE system in ergothioneine biosynthesis *via* constitutive expression. (a) Assembly of donor DNA based on the split-marker strategy. The donor DNA (8.2 kb for δ-site integration or 8.4 kb for rDNA-site integration) containing expression cassettes for *EGT1* from *C. purpurea* and *EGT2* from *N. crassa* is divided into three separate fragments for preparation, followed by assembly in vivo and expressed in *S. cerevisiae* BY4741 under strong constitutive promoters (*P*_*TDH3*_ and *P*_*CCW12*_, respectively). While *MET17*, the essential gene for sulfur metabolism, is chosen as the selectable marker regulated by the weak promoter *P*_*BTS1*_. (b) Yeast transformants after IMIGE screening. (c) Growth characteristics of yeast strains with varying integration copy numbers in 96-well plates, indicated by different amounts of cell accumulation at the well bottom. (d) Assessment of cell growth (OD_600_), ergothioneine titers, and integration copy numbers across transformants. The transformant expressing the *EGT1* and *EGT2* genes from an episomal plasmid served as the control.

In this study, plasmids containing the required components were constructed, and the donor DNA (Table S5), with a total length of 8.2 kb for the δ site integration or 8.4 kb for the rDNA site integration, was divided into three fragments and introduced using the split-marker strategy as outlined in Fig. 3. To enable ergothioneine synthesis, the enzymes Egt1 from *Claviceps purpurea* and Egt2 from *Neurospora crassa* were expressed in *S. cerevisiae* BY4741, under the regulation of the constitutive promoters *P*_*TDH3*_ and *P*_*CCW12*_, respectively. For multi-copy integration of *EGT1* and *EGT2*, both δ and rDNA sites were selected, with two sgRNAs designed for each site (Table S3). This setup led to the construction of four plasmids: pECas9-gRNA (Delta1/Delta2) and pECas9-gRNA (rDNA1/rDNA2). Methionine, whose synthesis was regulated by the weak promoter *P*_*BTS1*_, was used for selection; and the complementation of the *MET17* gene, which encodes a sulfur metabolism-related protein, enhanced sulfur metabolism in yeast cells, thereby promoting cysteine and methionine synthesis, thus providing sulfur for ergothioneine biosynthesis.

Ergothioneine biosynthesis was successfully adapted in *S. cerevisiae*, and increased EGT gene copy numbers led to enhanced production [25,26]. As shown in Fig. 3, after one round of screening, an 8-copy integrated strain eL1.1 was rapidly obtained, achieving an ergothioneine yield of 67.63 ± 9.27 mg/L after 120 h of liquid flask culture. This yield was 161.62 % higher than that of the control strain (episomal expression with plasmid pYES2-KlURA3-EGT1-EGT2) and 86.57 % higher than the best result from δ-site integration only (the 2-copy strain eδ1.1). Compared to first-generation strains in other studies [13,14,[26], [27], [28]], which lacked further metabolic flux optimization and/or fermentation improvement, this yield represents a significant enhancement.

By comparing the sizes of sedimented cells and copy numbers for the four strains in a 96-well plate, we observed that the colony size increased in the following order: eL1.3 < eL2.2 < eL1.1 < eL2.3 (Fig. 3). This pattern was consistent with the increase in the genomic integration copy numbers of the target genes, including *EGT1*, *EGT2*, and *MET17*. These findings validate the use of growth phenotypic recovery in the IMIGE system as a criterion for selecting high-copy integration strains and further support the correlation between *MET17* integration copy number and the growth of the engineered strains.

Using the IMIGE system, an 11-copy strain, eL2.3, was obtained after two cycles (5.5–6 days in total). However, both cell growth and ergothioneine production were significantly reduced. The OD_600_ of eL2.3 was only 7.41, representing a 52.10 % decrease compared to the 8-copy strain eL1.1, and the ergothioneine titer reached only 46.29 ± 2.46 mg/L. We hypothesize that this reduction is primarily due to the metabolic burden imposed by the high-copy expression of heterologous genes, which likely inhibits cell growth.

**Fig. 4:**
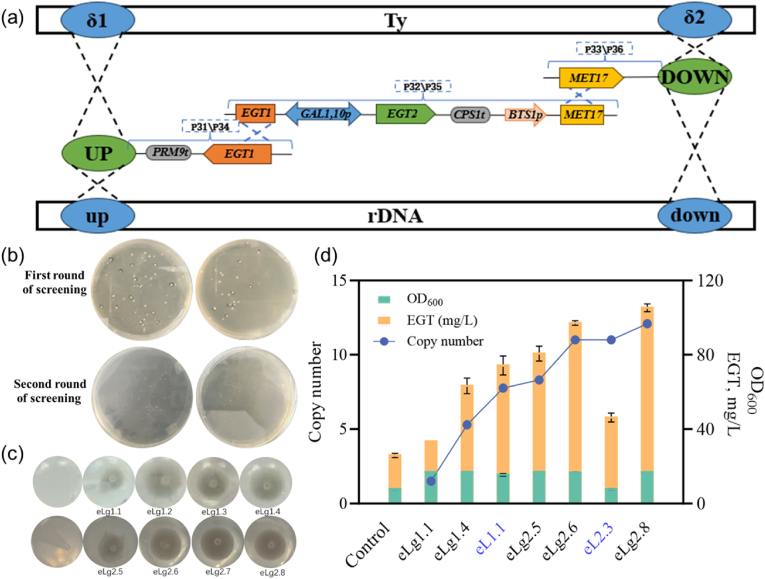
Application of the IMIGE system in ergothioneine biosynthesis *via* inducible expression. (a) Assembly of donor DNA based on the split-marker strategy. The donor DNA (7.5 kb for δ-site integration or 7.7 kb for rDNA-site integration) containing expression cassettes for *EGT1* from *C. purpurea* and *EGT2* from *N. crassa* is divided into three separate fragments for preparation, followed by assembly in vivo and expression in *S. cerevisiae* BY4741 under the galactose-inducible promoters *P*_*GAL1*_ and *P*_*GAL10*_. *MET17*, an essential gene involved in sulfur metabolism, is used as the selectable marker, regulated by the weak promoter *P*_*BTS1*_. (b) Yeast transformants after IMIGE screening. (c) Growth characteristics of yeast strains with varying integration copy numbers in 96-well plates, indicated by different amounts of cells at the well bottom. (d) Assessment of cell growth (OD_600_), ergothioneine titers, and integration copy numbers across transformants. The transformant expressing the *EGT1* and *EGT2* genes from an episomal plasmid served as the control.

To further investigate the cause of growth inhibition, we employed inducible promoters (*P*_*GAL1*_ and *P*_*GAL10*_) to drive the expression of *EGT1* and *EGT2* (Fig. 4). This strategy alleviated the growth suppression observed in high-copy strains with constitutive expression. The inducible strain eLg2.8, which carried 12 copies, exhibited normal cell growth (OD_600_ = 16.88) and produced an ergothioneine titer of 105.31 ± 1.53 mg/L, representing a 407.39 % increase compared to the control strain (episomal expression with plasmid) and 155.71 % to the highest-producing constitutive expression strain, eL1.1 (67.63 ± 9.27 mg/L). These results suggest that growth inhibition was primarily due to excessive heterologous enzyme expression, rather than the occupation of multicopy integration sites. Furthermore, our findings emphasize that, in addition to gene expression regulation, factors such as precursor availability, cofactor balance, and metabolic network efficiency are critical for maximizing ergothioneine biosynthesis [29]. The successful application of inducible expression further underscores the importance of dynamic metabolic control in optimizing both cell growth and product yield.

To assess the genetic stability of the engineered yeast strains, we tested the 8-copy strains (the constitutive expression strain eL1.1, and the inducible expression strain eLg2.5), as well as the control strain (episomal expression with plasmid) (Fig. S1). The strains were cultured in SC medium for 48 h, with serial passaging every 12 h, and fermentation continued for each generation until 144 h. The results showed that both 8-copy strains, eL1.1 and eLg2.5, maintained excellent genomic stability, with no reduction in gene copy number observed even after the fourth passage. While ergothioneine productivity (EGT/OD) showed slight fluctuations, these were likely due to differences in the culture system. In contrast, the plasmid-based control strain exhibited inconsistent qPCR results (data not provided), likely due to plasmid loss during passaging or segregation imbalance in progeny, which was reflected in significant fluctuations in ergothioneine productivity. These results demonstrate that high-copy integration strains generated *via* the IMIGE system are stable both genetically and in terms of ergothioneine production.

### 3.3 Construction of engineered yeast for cordycepin production based on the IMIGE system

**Fig. 5:**
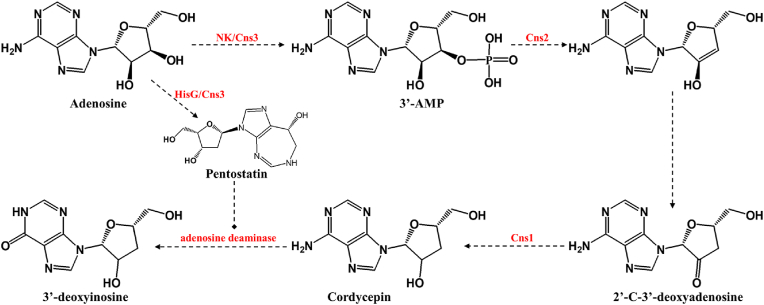
Putative biosynthetic pathway of cordycepin in *C. militaris*. The dual biosynthesis of cordycepin (COR) and pentostatin (PTN) is initiated by the enzyme Cns3, which contains two domains: an N-terminal nucleoside/nucleotide kinase (NK) domain and a C-terminal HisG family ATP phosphoribosyltransferase domain. The precursor adenosine is phosphorylated by the NK activity of Cns3 to form 3′-AMP, which is then hydrolyzed to 2′-C-3′-deoxyadenosine (2′-C-3′-dA) by Cns2, a metal-dependent phosphohydrolase. This intermediate is finally reduced to COR by the oxidoreductase Cns1. PTN production involves the HisG domain of Cns3, which inhibits adenosine deaminase (ADA) to prevent COR deamination to 3′-deoxyinosine.

Cordycepin, the primary bioactive compound in *Cordyceps militaris* and the first antibiotic nucleoside derived from a fungus, is highly valued in traditional Chinese medicine for its broad biological activities, including anti-tumor, antiviral, anti-inflammatory, antioxidant, and immunomodulatory effects [[30], [31], [32], [33], [34]]. With the completion of the whole-genome sequencing of *C. militaris*, Xia et al. proposed a biosynthetic pathway for cordycepin, beginning with adenosine and involving key enzymes Cns1-Cns3 (Fig. 5). Cns3 initiates the dual biosynthesis of cordycepin (COR) and pentostatin (PTN), while the formation of a Cns1-Cns2 complex is critical for COR production [15]. Microbial cell factories, supported by synthetic biology, are now emerging as a promising alternative for efficient and sustainable cordycepin production, offering superior yield and cost-effectiveness compared to traditional extraction methods [33].

**Fig. 6:**
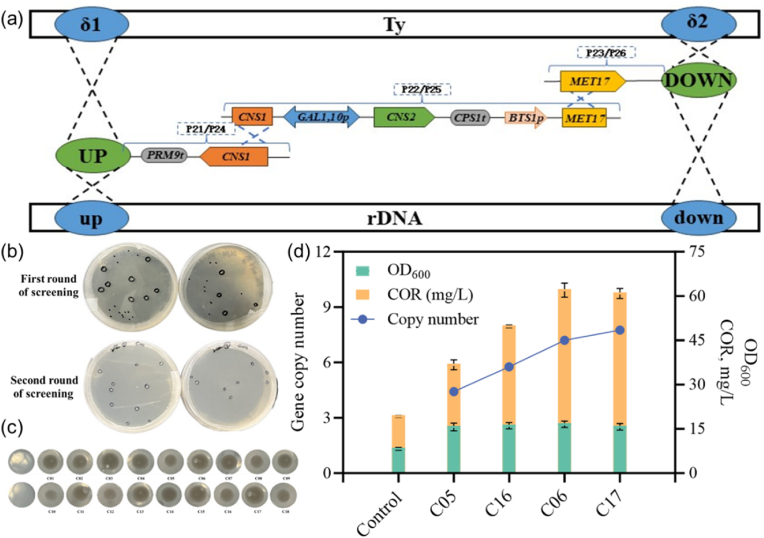
Application of IMIGE system in cordycepin biosynthesis *via* inducible expression. (a) Assembly of donor DNA based on the split-marker strategy. The donor DNA (6.7 kb for δ-site integration or 6.9 kb for rDNA-site integration) containing expression cassettes for *CNS1* and *CNS2* from *C. militaris* is divided into three separate fragments for preparation, followed by assembly in vivo and expressed in *S. cerevisiae* BY4741 under strong constitutive promoters (*P*_*TDH3*_ and *P*_*CCW12*_, respectively). While *MET17*, the essential gene for sulfur metabolism, is chosen as the selectable marker regulated by the weak promoter *P*_*BTS1*_. (b) Yeast transformants after IMIGE screening. (c) Growth characteristics of yeast strains with varying integration copy numbers in 96-well plates, indicated by different amounts of cells at the well bottom. (d) Assessment of cell growth (OD_600_), cordycepin titers, and integration copy numbers across transformants. The transformant expressing the *CNS1* and *CNS2* genes from an episomal plasmid served as the control.

In this context, the key enzymes for cordycepin biosynthesis, Cns1 and Cns2, were expressed in *S. cerevisiae* BY4741. The δ and rDNA genomic sites were selected for multi-copy integration of *CNS1* and *CNS2*, respectively. This process involved the construction of donor DNA (Table S5), with a total length of 6.7 kb for the δ site integration or 6.9 kb for the rDNA site integration, gRNA expression plasmids (pECas9-gRNA), the auxotrophic screening marker (*MET17*), and the use of the weak promoter *P*_*BTS1*_, following a strategy similar to that applied in the ergothioneine production process (Fig. 6).

Following multiple rounds of transformation and rapid iterative screening with the IMIGE system, four of the fastest-growing colonies were selected for further analysis. Due to the toxic effects of cordycepin accumulation in *S. cerevisiae*, the codon-optimized synthetic genes *CNS1* and *CNS2* were controlled by inducible promoters *P*_*GAL1*_ and *P*_*GAL10*_.

As illustrated in Fig. 6, the final cordycepin yield reached 62.01 ± 2.4 mg/L after two rounds of iterative screening, marking a 222.13 % increase compared to the control strain expressing *CNS1* and *CNS2 via* an episomal plasmid (19.25 ± 0.55 mg/L). The optimal copy number of *CNS1/CNS2* for maximum cordycepin yield was approximately 7, suggesting a correlation between gene copy number and yeast growth rate. Notably, further increase of the copy number (such as 8) did not enhance cordycepin production, similar to observations in ergothioneine biosynthesis. This result highlights the significance of managing product toxicity and optimizing metabolic flux in strain engineering [35], rather than relying solely on increasing target gene expression levels.

## 4 Conclusion

*S. cerevisiae* is a commonly used host for natural product biosynthesis, and multi-copy gene integration significantly enhances heterologous gene expression. However, traditional integration methods are often limited by challenges such as low gene copy numbers, lengthy screening processes, prolonged operational timelines, and high labor demands. To overcome these limitations, we developed the iterative multi-copy gene integration system (IMIGE), which combines the CRISPR/Cas9 system and the split-marker strategy. The IMIGE system involves three key steps: constructing Cas9-sgRNA expression vectors, preparing donor DNA fragments, and performing iterative transformations with rapid screening. Using this approach, engineered strains for ergothioneine and cordycepin production were successfully obtained within just two cycles (5.5–6 days), achieving yields of 105.31 ± 1.53 mg/L and 62.01 ± 2.4 mg/L, respectively, representing a significant increase compared to conventional single-copy integration or episomal plasmid expression methods. The system takes advantage of the δ and rDNA sequences in the yeast genome to achieve precise multi-copy integration, while using essential amino acid synthesis genes as selection markers to efficiently identify high-copy strains. Additionally, by enriching mixed cultures of transformants, the system minimizes labor-intensive steps such as colony picking and PCR validation, improving both strain retention and screening efficiency. The IMIGE system is a highly efficient, user-friendly tool that shows great promise for high-throughput screening and strain engineering, especially when paired with rapid product analysis methods.

## CRediT authorship contribution statement

**Ximei Chen:** Writing – original draft, Visualization, Methodology, Investigation, Formal analysis, Data curation. **Chenyang Li:** Writing – original draft, Visualization, Methodology, Investigation, Formal analysis, Data curation. **Xin Qiu:** Investigation, Data curation. **Ming Chen:** Writing – review & editing, Writing – original draft, Methodology. **Yongping Xu:** Writing – review & editing, Conceptualization. **Shuying Li:** Writing – review & editing, Conceptualization. **Qian Li:** Writing – review & editing, Writing – original draft, Funding acquisition, Conceptualization. **Liang Wang:** Writing – review & editing, Writing – original draft, Project administration, Funding acquisition, Conceptualization.

## Declaration of competing interest

Authors Yongping Xu and Shuying Li were employed by Dalian SEM Bio-Engineering Technology Co., Ltd., while Qian Li and Liang Wang are conducting postdoctoral research at the postdoctoral workstation of Dalian SEM Bio-Engineering Technology Co., Ltd. Other authors declare that they have no known competing financial interests or personal relationships that could have appeared to influence the work reported in the paper.

## References

[1] Li J.W., Vederas J.C. (2009). Drug discovery and natural products: end of an era or an endless frontier?. Science.

[2] Billingsley J.M., DeNicola A.B., Tang Y. (2016). Technology development for natural product biosynthesis in *Saccharomyces cerevisiae*. Curr Opin Biotechnol.

[3] Ro D.K., Paradise E.M., Ouellet M. (2006). Production of the antimalarial drug precursor artemisinic acid in engineered yeast. Nature.

[4] Luo X., Reiter M.A., d'Espaux L. (2019). Complete biosynthesis of cannabinoids and their unnatural analogues in yeast. Nature.

[5] Zhang J., Hansen L.G., Gudich O. (2022). A microbial supply chain for production of the anti-cancer drug vinblastine. Nature.

[6] Tang H., Lin S., Deng J. (2024). Engineering yeast for the de novo synthesis of jasmonates. Nat Synth.

[7] Liu Y., Zhao X., Gan F. (2024). Complete biosynthesis of QS-21 in engineered yeast. Nature.

[8] Jensen M.K., Keasling J.D. (2015). Recent applications of synthetic biology tools for yeast metabolic engineering. FEMS Yeast Res.

[9] Ting T.-Y., Li Y., Bunawan H. (2023). Current advancements in systems and synthetic biology studies of *Saccharomyces cerevisiae*. J Biosci Bioeng.

[10] Jacob O., van Lill G.R., den Haan R. (2022). CRISPR-based multi-gene integration strategies to create *Saccharomyces cerevisiae* strains for consolidated bioprocessing. Appl Sci.

[11] Shi S., Liang Y., Zhang M.M. (2016). A highly efficient single-step, markerless strategy for multi-copy chromosomal integration of large biochemical pathways in *Saccharomyces cerevisiae*. Metab Eng.

[12] Kobayashi T., Ganley A.R. (2005). Recombination regulation by transcription-induced cohesin dissociation in rDNA repeats. Science.

[13] Doyle S., Cuskelly D.D., Conlon N. (2022). A single *Aspergillus fumigatus* gene enables ergothioneine biosynthesis and secretion by *Saccharomyces cerevisiae*. Int J Mol Sci.

[14] van der Hoek S.A., Darbani B., Zugaj K.E. (2019). Engineering the yeast *Saccharomyces cerevisiae* for the production of L-(+)-ergothioneine. Front Bioeng Biotechnol.

[15] Xia Y., Luo F., Shang Y. (2017). Fungal cordycepin biosynthesis is coupled with the production of the safeguard molecule pentostatin. Cell Chem Biol.

[16] Lõoke M., Kristjuhan K., Kristjuhan A. (2011). Extraction of genomic DNA from yeasts for PCR-based applications. Biotechniques.

[17] Qi H., Yu L., Li Y. (2022). Developing multi-copy chromosomal integration strategies for heterologous biosynthesis of caffeic acid in *Saccharomyces cerevisiae*. Front Microbiol.

[18] Xie W., Ye L., Lv X. (2015). Sequential control of biosynthetic pathways for balanced utilization of metabolic intermediates in *Saccharomyces cerevisiae*. Metab Eng.

[19] Nambu-Nishida Y., Sakihama Y., Ishii J. (2018). Selection of yeast *Saccharomyces cerevisiae* promoters available for xylose cultivation and fermentation. J Biosci Bioeng.

[20] Liu H.M., Tang W., Wang X.Y. (2023). Safe and effective antioxidant: the biological mechanism and potential pathways of ergothioneine in the skin. Molecules.

[21] Xiong K., Xue S., Guo H. (2024). Ergothioneine: new functional factor in fermented foods. Crit Rev Food Sci Nutr.

[22] Han Y., Tang X., Zhang Y. (2021). The current status of biotechnological production and the application of a novel antioxidant ergothioneine. Crit Rev Biotechnol.

[23] Seebeck F.P. (2010). In vitro reconstitution of mycobacterial ergothioneine biosynthesis. J Am Chem Soc.

[24] Stampfli A.R., Blankenfeldt W., Seebeck F.P. (2020). Structural basis of ergothioneine biosynthesis. Curr Opin Struct Biol.

[25] Takusagawa S., Satoh Y., Ohtsu I. (2019). Ergothioneine production with *Aspergillus oryzae*. Biosci Biotechnol Biochem.

[26] van der Hoek S.A., Rusnák M., Wang G. (2022). Engineering precursor supply for the high-level production of ergothioneine in *Saccharomyces cerevisiae*. Metab Eng.

[27] Yu Y.H., Pan H.Y., Guo L.Q. (2020). Successful biosynthesis of natural antioxidant ergothioneine in *Saccharomyces cerevisiae* required only two genes from *Grifola frondosa*. Microb Cell Fact.

[28] Yu Y.H., Liu H.Q., Zhang S. (2024). Highly efficient production of ergothioneine from glycerol through gene mining and CRISPR-Cas9 synthetic biology technology in *Saccharomyces cerevisiae*. ACS Sustainable Chem Eng.

[29] Du F., Li Z., Li X. (2024). Optimizing multicopy chromosomal integration for stable high-performing strains. Nat Chem Biol.

[30] Songprakhon P., Panya A., Choomee K. (2024). Cordycepin exhibits both antiviral and anti-inflammatory effects against dengue virus infection. iScience.

[31] Panya A., Songprakhon P., Panwong S. (2021). Cordycepin inhibits virus replication in dengue virus-infected vero cells. Molecules.

[32] Jeong J.W., Jin C.Y., Kim G.Y. (2010). Anti-inflammatory effects of cordycepin via suppression of inflammatory mediators in BV2 microglial cells. Int Immunopharmacol.

[33] Kunhorm P., Chaicharoenaudomrung N., Noisa P. (2019). Enrichment of cordycepin for cosmeceutical applications: culture systems and strategies. Appl Microbiol Biotechnol.

[34] Khan M.A., Tania M. (2020). Cordycepin in anticancer research: molecular mechanism of therapeutic effects. Curr Med Chem.

[35] Wang H., Fu X., Zuo X. (2024). Overproduction of cordycepin in *Saccharomyces cerevisiae* by cordycepin synthase screening and metabolic engineering. AIChE J.

