# Supplementary material for: CRISPR/Cas9-based iterative multi-copy integration for improved metabolite yields in Saccharomyces cerevisiae
Source: Synth Syst Biotechnol. 2025 Mar 5;10(2):629–37. doi: 10.1016/j.synbio.2025.02.016 (PMC11946509; doi:10.1016/j.synbio.2025.02.016)
Supplement: Multimedia component 1 [file mmc1.docx]

CRISPR/Cas9-based Iterative Multi-Copy Integration for Improved Metabolite Yields in *Saccharomyces cerevisiae*

Ximei Chen^a,1^, Chenyang Li^b,1^, Xin Qiu^b^, Ming Chen^a^, Yongping Xu^c,d^, Shuying Li^c^, Qian Li^b,c,*^, and Liang Wang^a,c,*^

*^a^**School of Biological Engineering, Dalian Polytechnic University, Dalian 116034, China.*

*^b^School of Life and Health, Dalian University, Dalian 116622, China.*

*^c^Postdoctoral Workstation of Dalian SEM Bio-Engineering Technology Co. Ltd., Dalian 116000, China*

*^d^School of Bioengineering, Dalian University of Technology, Dalian 116024, China*

***Corresponding Authors:**

Liang Wang, School of Biological Engineering, Dalian Polytechnic University, No.1 Qing-gong-yuan, Ganjingzi District, Dalian City 116034, China; E-mail address: wangliang@dlpu.edu.cn;

Qian Li, Department of Life and Health, Dalian University, No. 2 Xuefu Street, Dalian Economic and Technological Development Zone, Dalian City 116622, China; E-mail address: liqian@dlu.edu.cn.

^1^These authors contributed equally to this work: Ximei Chen, Chenyang Li.

**Supplementary Tables and Figures**

**Tab. S1 Strains and plasmids**

| **Strain/Plasmid** | **Description** | **Source** |
| --- | --- | --- |
| **Strains** |  |  |
| *E. coli* DH5α | For preparation of competent cells | Our lab |
| *S. cerevisiae* BY4741 | MATa*, ura3-52*, *leu2*, *his3Δ1*, *Δmet17* | Our lab |
| eδ1.1 | *S. cerevisiae* BY4741, multi-copy integration of the biosynthetic pathway of ergothioneine in δ elements | This study |
| eL1.1-2.3 | *S. cerevisiae* BY4741, multi-copy integration of the biosynthetic pathway of ergothioneine in both δ elements and rDNA regions, with the heterologous expression of *EGT1* and *EGT2* driven by constitutive promoters *P_TDH3_* and *P_CCW12_*, respectively. | This study |
| eLg1.1-2.8 | *S. cerevisiae* BY4741, multi-copy integration of the biosynthetic pathway of ergothioneine in both δ elements and rDNA regions, with the heterologous expression of *EGT1* and *EGT2* driven by inducible promoters *P_GAL1_* and *P_GAL10_*, respectively. | This study |
| C01-C18 | *S. cerevisiae* BY4741, multi-copy integration of the biosynthetic pathway of cordycepin in both δ elements and rDNA regions, with the heterologous expression of *CNS1* and *CNS2* driven by inducible promoters *P_GAL1_* and *P_GAL10_*, respectively. | This study |
| **Plasmids** |  |  |
| pYES2-*KlURA3*-*EGT*1-*EGT*2 | Amp^r^, *URA*3, with the expression cassettes for ergothioneine biosynthesis | This study |
| pYES2-*CNS*1-*CNS*2 | Amp^r^, *URA*3, with expression cassettes for  cordycepin biosynthesis | This study |
| pECAS9-KlURA3-gRNA | Amp^r^, *URA*3, with Cas9 and gRNA | Donated by Prof. Zhiwei Zhu from Dalian University of Technology |
| pYES2-URA3-MET17 | Amp^r^, *URA*3, for cloning of *MET17* | Our lab |
| pCas9-gRNA（Delta1） | Amp^r^, *URA*3, with gRNA1 targeting δ elements | This study |
| pCas9-gRNA（Delta2） | Amp^r^, *URA*3, with gRNA2 targeting δ elements | This study |
| pCas9-gRNA（rDNA1） | Amp^r^, *URA*3, with gRNA3 targeting rDNA regions | This study |
| pCas9-gRNA（rDNA2） | Amp^r^, *URA*3, with gRNA4 targeting rDNA regions | This study |

**Tab. S2 Primers used in this study.**

| **Primers** | **Sequences (5’-3’)** | **Description** |
| --- | --- | --- |
| **Constructing the Cas9-sgRNA expression vectors** | | |
| F100 | GTAGTGCCCTCTTGGGCTAGCGGTAAAGGTGCGCATTTTTTCA | Overlap of the fragments of pECAS9-KlURA3-gRNA |
| R100 | AAGCACAGATGCTTCGTTAACAAAGATATGCTATTGAAGTGCAAGATG |  |
| F101 | CCAGTTTTTCGCTAACTACCTGTTTTAGAGCTAGAAATAGCAAGTTAAAATAAG | gRNA1 targeting δ elements |
| R101 | CAGGTAGTTAGCGAAAAACTGGATCATTTATCTTTCACTGCGGAGAAG |  |
| F102 | CCAATTGCGATTTTCGAACACGTTTTAGAGCTAGAAATAGCAAGTTAAAATAAG | gRNA3 targeting δ elements |
| R102 | CGTGTTCGAAAATCGCAATTGGATCATTTATCTTTCACTGCGGAGAAG |  |
| F103 | CATGCCCCCTGGAATACCAAGGTTTTAGAGCTAGAAATAGCAAGTTAAAATAAG | gRNA4 targeting rDNA regions |
| R103 | CCTTGGTATTCCAGGGGGCATGATCATTTATCTTTCACTGCGGAGAAG |  |
| F104 | CCCGCTTATTGATATGCTTAAGTTTTAGAGCTAGAAATAGCAAGTTAAAATAAG | gRNA2 targeting rDNA2 regions |
| R104 | CTTAAGCATATCAATAAGCGGGATCATTTATCTTTCACTGCGGAGAAG |  |
| **Preparing linear donor DNA fragments for EGT and COR biosynthesis** | | |
| F201 | TTGTTGGAATAAAAATCAACTATCATCTAC | the upstream homology arm of the δ elements (P201) |
| R201 | GAAAATACGATGTTGAAAATAAAAGGGAATCTGCAATTCTACAC |  |
| F202 | TGTCAAATCAAGTGTCAAATGGCTGGCAACTAATAGGGACAC | the downstream homology arm of the δ elements (P202) |
| R202 | AGGCTATAATATCAGGTATACAGAATATACTAGAAG |  |
| F203 | TCTATGACGTCCTGTTCCAAGG | the upstream homology arm of the rDNA region (P203) |
| R203 | GAAAATACGATGTTGAAAATGCAAGTACGGTCGTTTTAGG |  |
| F204 | TGTCAAATCAAGTGTCAAATATTATACCTCAAGCACGCAG | the downstream homology arm of the rDNA region (P204) |
| R204 | GGCCCAGAGGTAACAAACAC |  |
| F211 | AGAATTGCAGATTCCCTTTTATTTTCAACATCGTATTTTCCGAAGCGT | the expression cassette of the key gene *EGT*2 (P211) |
| R211 | GGAGTGAGTAGAGATGGCGGCACCCATGAACCACACGGTTAG |  |
| F212 | AACCGTGTGGTTCATGGGTGCCGCCATCTCTACTCACTC | the promoter *P_BTS1_* (P212) |
| R212 | GTATCGAAATGAGATGGCATTGATTTTCCAGACTCGTAAAC |  |
| F213 | TTTACGAGTCTGGAAAATCAATGCCATCTCATTTCGATACTG | *MET17* (P213) |
| R213 | ATAATGATAAACTCGAACTGTCATGGTTTTTGGCCAGCGAAAAC |  |
| F214 | TCGCTGGCCAAAAACCATGACAGTTCGAGTTTATCATTATCAATACTGCC | the expression cassette of the key gene *EGT1* (P214) |
| R214 | GTGTCCCTATTAGTTGCCAGCCATTTGACACTTGATTTGACACTTC |  |
| Fg201 | TTGTTGGAATAAAAATCAACTATCATCTAC | the upstream homology  arm of the δ elements  (Pg201) |
| Rg201 | GAAAATACGATGTTGAAAATAAAAGGGAATCTGCAATTCTACAC |  |
| Fg202 | TCGCTGGCCAAAAACCATGAGGCTGGCAACTAATAGGGACAC | the downstream  homology arm of the δ  elements (Pg202) |
| Rg202 | AGGCTATAATATCAGGTATACAGAATATACTAGAAG |  |
| Fg203 | TCTATGACGTCCTGTTCCAAGG | the upstream homology  arm of the rDNA region  (Pg203) |
| Rg203 | GAAAATACGATGTTGAAAATGCAAGTACGGTCGTTTTAGG |  |
| Fg204 | TCGCTGGCCAAAAACCATGAATTATACCTCAAGCACGCAGAGAAAC | the downstream  homology arm of the  rDNA region (Pg204) |
| Rg204 | GGCCCAGAGGTAACAAACAC |  |
| Fg211 | ATTTTCAACATCGTATTTTCCGAAGCGT | the expression cassette  of the key gene *EGT*1(Pg211) |
| Rg211 | AAAAGTAAGAATTTTTGAAAATGGGTTTATTAGAAGGTGAAGAATTAG |  |
| Fg212 | TCACCTTCTAATAAACCCATTTTCAAAAATTCTTACTTTTTTTTTGGATGGAC | the promoter P*_GAL_*_1,10_  (Pg212) |
| Rg212 | ATAGATTCAGCAGATGGCATGTTTTTCTCCTTGACGTTAAAGTATAGAG |  |
| Fg213 | TAACGTCAAGGAGAAAAAACATGCCATCTGCTGAATCTATGAC | the expression cassette  of the key gene *EGT*2  (Pg213) |
| Rg213 | GGAGTGAGTAGAGATGGCGGATTTGACACTTGATTTGACACTTC |  |
| Fg214 | TGTCAAATCAAGTGTCAAATCCGCCATCTCTACTCACTCC | the promoter P*_BTS_*_1_ (Pg214) |
| Rg214 | GTATCGAAATGAGATGGCATTGATTTTCCAGACTCGTAAAC |  |
| Fg215 | TTTACGAGTCTGGAAAATCAATGCCATCTCATTTCGATACTG | *MET*17 (Pg215) |
| Rg215 | GTCCCTATTAGTTGCCAGCCTCATGGTTTTTGGCCAGC |  |
| F301 | TTGTTGGAATAAAAATCAACTATCATCTAC | the upstream homology arm of the δ element (P301) |
| R301 | GAAAATACGATGTTGAAAATAAAAGGGAATCTGCAATTCTACAC |  |
| F302 | TCGCTGGCCAAAAACCATGAGGCTGGCAACTAATAGGGACAC | the downstream homology arm of the δ element (P302) |
| R302 | AGGCTATAATATCAGGTATACAGAATATACTAGAAG |  |
| F303 | TCTATGACGTCCTGTTCCAAGG | the upstream homology arm of the rDNA region (P303) |
| R303 | GAAAATACGATGTTGAAAATGCAAGTACGGTCGTTTTAGG |  |
| F304 | TCGCTGGCCAAAAACCATGAATTATACCTCAAGCACGCAGAGAAAC | the downstream homology arm of the rDNA region (P304) |
| R304 | GGCCCAGAGGTAACAAACAC |  |
| F311 | AGAATTGCAGATTCCCTTTTATTTTCAACATCGTATTTTCCGAAGCGT | the terminator T*_PRM9_* (P311) |
| R311 | AGGTTGGTATCGCCTAATAGACAGAAGACGGGAGACACTAGC |  |
| F312 | TAGTGTCTCCCGTCTTCTGTCTATTAGGCGATACCAACCTTGGAAC | *CNS1* (P312) |
| R312 | AAAAGTAAGAATTTTTGAAAATGGCCATGAACGAAAACGG |  |
| F313 | CCGTTTTCGTTCATGGCCATTTTCAAAAATTCTTACTTTTTTTTTGGATGGACGC | the promoter P*_GAL1,10_* (P313) |
| R313 | GCAGAAGTTGGACAGGACATGTTTTTTCTCCTTGACGTTAAAGTATAGAGGTATATTAAC |  |
| F314 | TAACGTCAAGGAGAAAAAACATGTCCTGTCCAACTTCTGCTGG | *CNS2* (P314) |
| R314 | TGACTATTCAATCATTGCGCTTACTATCTGTGTTGAGTCCTGGACAAGATG |  |
| F315 | GGACTCAACACAGATAGTAAGCGCAATGATTGAATAGTCAAAG | the terminator T*_CPS1_* (P315) |
| R315 | GGAGTGAGTAGAGATGGCGGATTTGACACTTGATTTGACACTTC |  |
| F316 | TGTCAAATCAAGTGTCAAATCCGCCATCTCTACTCACTCC | the promoter P*_BTS1_* (P316) |
| R316 | GTATCGAAATGAGATGGCATTGATTTTCCAGACTCGTAAAC |  |
| F317 | TTTACGAGTCTGGAAAATCAATGCCATCTCATTTCGATACTG | *MET17* (P317) |
| R317 | GTCCCTATTAGTTGCCAGCCTCATGG |  |
| F201 | TTGTTGGAATAAAAATCAACTATCATCTAC | the fragment of P11 |
| R213 | ATAATGATAAACTCGAACTGTCATGGTTTTTGGCCAGCGAAAAC |  |
| F213 | TTTACGAGTCTGGAAAATCAATGCCATCTCATTTCGATACTG | the fragment of P12 |
| R214 | GTGTCCCTATTAGTTGCCAGCCATTTGACACTTGATTTGACACTTC |  |
| F214 | TCGCTGGCCAAAAACCATGACAGTTCGAGTTTATCATTATCAATACTGCC | the fragment of P13 |
| R202 | AGGCTATAATATCAGGTATACAGAATATACTAGAAG |  |
| F203 | TCTATGACGTCCTGTTCCAAGG | the fragment of P14 |
| R213 | ATAATGATAAACTCGAACTGTCATGGTTTTTGGCCAGCGAAAAC |  |
| F213 | TTTACGAGTCTGGAAAATCAATGCCATCTCATTTCGATACTG | the fragment of P15 |
| R214 | GTGTCCCTATTAGTTGCCAGCCATTTGACACTTGATTTGACACTTC |  |
| F214 | TCGCTGGCCAAAAACCATGACAGTTCGAGTTTATCATTATCAATACTGCC | the fragment of P16 |
| R204 | GGCCCAGAGGTAACAAACAC |  |
| Fg201 | TTGTTGGAATAAAAATCAACTATCATCTAC | the fragment of P31 |
| Rg211 | AAAAGTAAGAATTTTTGAAAATGGGTTTATTAGAAGGTGAAGAATTAG |  |
| Fg211 | ATTTTCAACATCGTATTTTCCGAAGCGT | the fragment of P32 |
| Rg215 | GTCCCTATTAGTTGCCAGCCTCATGGTTTTTGGCCAGC |  |
| Fg215 | TTTACGAGTCTGGAAAATCAATGCCATCTCATTTCGATACTG | the fragment of P33 |
| Rg202 | AGGCTATAATATCAGGTATACAGAATATACTAGAAG |  |
| Fg203 | TCTATGACGTCCTGTTCCAAGG | the fragment of P34 |
| Rg211 | AAAAGTAAGAATTTTTGAAAATGGGTTTATTAGAAGGTGAAGAATTAG |  |
| Fg211 | ATTTTCAACATCGTATTTTCCGAAGCGT | the fragment of P35 |
| Rg215 | GTCCCTATTAGTTGCCAGCCTCATGGTTTTTGGCCAGC |  |
| Fg215 | TTTACGAGTCTGGAAAATCAATGCCATCTCATTTCGATACTG | the fragment of P36 |
| Rg204 | GGCCCAGAGGTAACAAACAC |  |
| F301 | TTGTTGGAATAAAAATCAACTATCATCTAC | the fragment of P21 |
| R312 | AAAAGTAAGAATTTTTGAAAATGGCCATGAACGAAAACGG |  |
| F312 | TAGTGTCTCCCGTCTTCTGTCTATTAGGCGATACCAACCTTGGAAC | the fragment of P22 |
| R317 | GTCCCTATTAGTTGCCAGCCTCATGG |  |
| F317 | TTTACGAGTCTGGAAAATCAATGCCATCTCATTTCGATACTG | the fragment of P23 |
| R302 | AGGCTATAATATCAGGTATACAGAATATACTAGAAG |  |
| F303 | TCTATGACGTCCTGTTCCAAGG | the fragment of P24 |
| R312 | AAAAGTAAGAATTTTTGAAAATGGCCATGAACGAAAACGG |  |
| F312 | TAGTGTCTCCCGTCTTCTGTCTATTAGGCGATACCAACCTTGGAAC | the fragment of P25 |
| R317 | GTCCCTATTAGTTGCCAGCCTCATGG |  |
| F317 | TTTACGAGTCTGGAAAATCAATGCCATCTCATTTCGATACTG | the fragment of P26 |
| R304 | GGCCCAGAGGTAACAAACAC |  |

**Tab. S3 Sequences of sgRNA used in this study.**

| Name | sgRNA sequence | Description |
| --- | --- | --- |
| sgRNA-1 | CAGTTTTTCGCTAACTACCTgttttagagctagaaatagcaagttaaaataaggctagtccgttatcaacttgaaaaagtggcaccgagtcggtg | pCas9-gRNA（Delta1） |
| sgRNA-2 | CAATTGCGATTTTCGAACACgttttagagctagaaatagcaagttaaaataaggctagtccgttatcaacttgaaaaagtggcaccgagtcggtg | pCas9-gRNA（Delta2） |
| sgRNA-3 | ATGCCCCCTGGAATACCAAGgttttagagctagaaatagcaagttaaaataaggctagtccgttatcaacttgaaaaagtggcaccgagtcggtg | pCas9-gRNA （rDNA1） |
| sgRNA-4 | CCGCTTATTGATATGCTTAAgttttagagctagaaatagcaagttaaaataaggctagtccgttatcaacttgaaaaagtggcaccgagtcggtg | pCas9-gRNA （rDNA2） |

**Tab. S4 Homologous arm sequences for the δ and rDNA sites used in this study.**

| Name | sequence |
| --- | --- |
| The upstream homologous arm for the δ genomic region (UP-δ region) | TTGTTGGAATAAAAATCAACTATCATCTACTAACTAGTATTTACGTTACTAGTATATTATCATATACGGTGTTAGAAGATGACGCAAATGATGAGAAATAGTCATCTAAATTAGTGGAAGCTGAAACGCAAGGATTGATAATGTAATAGGATCAATGAATATTAACATATAAAATGATGATAATAATATTTATAGAATTGTGTAGAATTGCAGATTCCCTTTT |
| The downstream homologous arm for the δ genomic sites (DOWN-δ region) | GGCTGGCAACTAATAGGGACACTACCAATATATTATCATATACGGTGTTAGACGATGACATAAGATACGAGGAACTGTCATCGAAGTTAGAGGAAGCTGAAATGCAAGGATTGATAATGTAATAGGATAATGAAACATATAAAACGGAATGAGGAATAATCGTAATATTAGTATATAGAGATAAAGATTCCATTTTGAGGATTCCTATATCCTCGAGGAGAACTTCTAGTATATTCTGTATACCTGATATTATAGCCT |
| The upstream homologous arm for the rDNA genomic sites (UP-rDNA region) | TCTATGACGTCCTGTTCCAAGGAACATAGACAAGGAACGGCCCCAAAGTTGCCCTCTCCAAATTACAACTCGGGCACCGAAGGTACCAGATTTCAAATTTGAGCTTTTGCCGCTTCACTCGCCGTTACTAAGGCAATCCCGGTTGGTTTCTTTTCCTCCGCTTATTGATATGCTTAAGTTCAGCGGGTACTCCTACCTGATTTGAGGTCAAACTTTAAGAACATTGTTCGCCTAGACGCTCTCTTCTTATCGATAACGTTCCAATACGCTCAGTATAAAAAAAGATTAGCCGCAGTTGGTAAAACCTAAAACGACCGTACTTGC |
| The downstream homologous arm for the rDNA genomic sites (DOWN-rDNA region) | ATTATACCTCAAGCACGCAGAGAAACCTCTCTTTGGAAAAAAAACATCCAATGAAAAGGCCAGCAATTTCAAGTTAACTCCAAAGAGTATCACTCACTACCAAACAGAATGTTTGAGAAGGAAATGACGCTCAAACAGGCATGCCCCCTGGAATACCAAGGGGCGCAATGTGCGTTCAAAGATTCGATGATTCACGGAATTCTGCAATTCACATTACGTATCGCATTTCGCTGCGTTCTTCATCGATGCGAGAACCAAGAGATCCGTTGTTGAAAGTTTTTAATATTTTAAAATTTCCAGTTACGAAAATTCTTGTTTTTGACAAAAATTTAATGAATAGATAAAATTGTTTGTGTTTGTTACCTCTGGGCC |

**Tab. S5 Sequences of the donor DNA used in this study.**

| Name/ Description | Sequence |
| --- | --- |
| donor DNA-1  (multi-copy integration at δ site for EGT biosynthesis, with constitutive expression of *EGT1* and *EGT2*) | TTGTTGGAATAAAAATCAACTATCATCTACTAACTAGTATTTACGTTACTAGTATATTATCATATACGGTGTTAGAAGATGACGCAAATGATGAGAAATAGTCATCTAAATTAGTGGAAGCTGAAACGCAAGGATTGATAATGTAATAGGATCAATGAATATTAACATATAAAATGATGATAATAATATTTATAGAATTGTGTAGAATTGCAGATTCCCTTTTATTTTCAACATCGTATTTTCCGAAGCGTTGCATAATGAAAATGTGATAATAAGGCAGTGAGCTACTTTGTCACAACAAAGTACGAGAACAGATGAGGTTCCGTGGTGTTTTTGGGGCAACCGCACTATTTGGAGCGCTGTATATATTTATATCCTACTACAAAAAATCATAAATGACACTGAATTGGACACATGCTAGCGTCAAATACCTTGCCTGGTAAAGTTGTGTGCTAGTGTCTCCCGTCTTCTGTGAGCTCTTATTCAACTGGTTGTGGAACTAAATATTCCCTCCTCCTAATCCTTTCACACAAAGCCTTTAAAATATCACCAGCTGCTTCATAATCTTGTTCATCTAAATAAATTTGAGCTGATAACCTAACCCAATATCTATCTTGCATAACAAACAATGTCATAAAAGTTTTATAATCATCAAATAACCTTTCTCTCATCCATTTATCAACTAAAGCAACATTTTCCCTAGCAACAACAACATCAGGTGCAGCAATTGCAGCAGATGGTGCTGCATCCAATGCAGTAGAAGCATCTTCATCATCAACTCTCATAGGTAAAGCAACATTACCCATAGCACAATTAGTTAAAGTTTCTGTTTCATTATCCAAATGAGTAGTACCCAATGCCCTAGCAACAATCCTAATACCTTTTTTATTTAATGCCCACAAATACCTTAAAATATTTTCTTCACCACCACAAACTCTTTCCCTCCATGCAATTGCATCAGCAACACACAAATATGGACCATTATCTCTAGTACCAACAAATTCAAAATTAGCAACATAGCGGGATTTAGAACCAGGTGGTGCAGGAGCTGCTGAAGGTGGAACATAACCATGAGATGTAGCCAATGCTGTCCTTAATAAACATTGAGTCCTAGCTGGTGTATATAACATTGCACAACCCCTAGGAACTAATAACCATTTATGACAATTAGAAACAAAAAAATCAGGATCGGCTGCTGTTAAATCTAATCTAACCATACCAACACCTTGTGCACCATCAACTAAAGATAAAATACCCAATTCTCTACAAACTCTAACCGCTGCTTCCCAAGGAAAAACAACACCTGGCCTTGAAGTAACAACATCCATCATAGCCAATCTTGCCCTTTTACCTTCTCTAGCAACTTGTGTTGCTGCACCCCTCAAAGCTGCAATAACATCAGCATCTTCAACTGGATATTCCAATTCAATAGTCCTATGTTCAACTTTACCTGCAAAATATTCAACAATATAATCAGCAGCATTACCACATGCTTCATAAACTGTAGAAAAAGACAAAATAACATCTTTTTGACCACCTTTTTCCAAAGAATCCCACCTCAAATTCCTCAAAACTGTATTAACACCTTCAGTTGCATTACCAACAAAAACAACTGTATCTAATGGAGCATTAACAATTTTAGCAACCGCCGCTCGACTCCTATGTAATAATTTAGATTCTTCATACCTAATAAAATGATCAGGCCTAGCTTCTGCTTGATCTTGATATGCTCTTAATTTATCCCTAATATACAATGGATAAGTACCAAATGAACCATGATTTAAATTCCTCCATGCTGGATCAAATAAAAATTCTGATTTCCAAGCTCTACCAAATGCCAAAACTTCCCTTTGTGGAGTTTCTTGTAAAGGTTCTGATTCTGCTTCCAATTTATGTTCTGGTTCTTTCCTAGGACCCAATTGACCTTCAGTTTCTGGTTCACCATCAGGAGCCCTTTCAGGAACATGTTCCAATTTTAATTCTGGTTCTCTTTCTGGCCTAGGTTCACCACCTTGACCCCTACCCCTCAAAACTAATTCTTCACCTTCTAATAAACCCATTGTTTTGAGCTCTATTGATATAGTGTTTAAGCGAATGACAGAAGATTAATTTCTTGGTATGTTAGGAAAGAATAAAGGAGAATAAGAATAATTAGAACAATGTAGGATGGAAAGAAAGATTATCAAGCATGCCGACTTTATATACTTGAACGGAGGCAAAGGATGCAAAATTTTCTCACATTTCTTTCTGCCGTTATGTTGGAAGTAAGACTCCCATTATCGCAATACTGCAACACGAATATGCAAAATTTGCTGAGTTATCGCAGATAGTTGTTGCAAAGATAGCGGCGTAGGTGGCCGCGAAATGGGGAATTCCAAAACAAACGGTTTTTTTACTCCTGAGAAATACTTGTACGGGATAATCCAGGGCCTACCACCCACGCTTCGAGGATTGGCTTTTATTTTTTTTTTTTTGGTGGCGTTTTATTTCTTTCCCGCTTTCTGGGACTTGTGCGGAGTTTTGAGAGGGGCGCGCGGCAAAGGATTCCCAAAACGGAAATCAGACGCCAATAGCCAGCACTCAAAGCAGTTCTGGACCCATTCCGATTTTCCCATTTGGTTCTTGCGCGTGCTGATTCCGACACGCGCGTCTATAAATAGCATGAAGTATCCGCACACCGCAGCGTTAGTGAGGTGAGGGTGGCAGCAAGCTAATTCCCGCATCTGGAATCTGAACTGCCCCTTTTGGACTAACCGTGTGGTTCATGGGTGCCGCCATCTCTACTCACTCCATAATATTACATATAGATATAGGACAAGCCCGCATTTTCATACTGAAAGGTAAACTTCTATTATTATAGTGGTATCCAACGTTCACCGCTTCCAGCATAGCAGAAATTACGTGTTTTTGCATATGTTATGCTGATCATTGTATGCTTACTACCATTTTTCTTTGCTTCGCCTTGCCTTCTTTGACGTTTTTTTGAAGCAAAAAAAAAGTCAAGACAGATGTGCTTACAAAACCATGTAAGGCTCATTTTCAAAGAAGCTACTAATAGAAAGAGAACAAAGCGTTTACGAGTCTGGAAAATCAATGCCATCTCATTTCGATACTGTTCAACTACACGCCGGCCAAGAGAACCCTGGTGACAATGCTCACAGATCCAGAGCTGTACCAATTTACGCCACCACTTCTTATGTTTTCGAAAACTCTAAGCATGGTTCGCAATTGTTTGGTCTAGAAGTTCCAGGTTACGTCTATTCCCGTTTCCAAAACCCAACCAGTAATGTTTTGGAAGAAAGAATTGCTGCTTTAGAAGGTGGTGCTGCTGCTTTGGCTGTTTCCTCCGGTCAAGCCGCTCAAACCCTTGCCATCCAAGGTTTGGCACACACTGGTGACAACATCGTTTCCACTTCTTACTTATACGGTGGTACTTATAACCAGTTCAAAATCTCGTTCAAAAGATTTGGTATCGAGGCTAGATTTGTTGAAGGTGACAATCCAGAAGAATTCGAAAAGGTCTTTGATGAAAGAACCAAGGCTGTTTATTTGGAAACCATTGGTAATCCAAAGTACAATGTTCCGGATTTTGAAAAAATTGTTGCAATTGCTCACAAACACGGTATTCCAGTTGTCGTTGACAACACATTTGGTGCCGGTGGTTACTTCTGTCAGCCAATTAAATACGGTGCTGATATTGTAACACATTCTGCTACCAAATGGATTGGTGGTCATGGTACTACTATCGGTGGTATTATTGTTGACTCTGGTAAGTTCCCATGGAAGGACTACCCAGAAAAGTTCCCTCAATTCTCTCAACCTGCCGAAGGATATCACGGTACTATCTACAATGAAGCCTACGGTAACTTGGCATACATCGTTCATGTTAGAACTGAACTATTAAGAGATTTGGGTCCATTGATGAACCCATTTGCCTCTTTCTTGCTACTACAAGGTGTTGAAACATTATCTTTGAGAGCTGAAAGACACGGTGAAAATGCATTGAAGTTAGCCAAATGGTTAGAACAATCCCCATACGTATCTTGGGTTTCATACCCTGGTTTAGCATCTCATTCTCATCATGAAAATGCTAAGAAGTATCTATCTAACGGTTTCGGTGGTGTCTTATCTTTCGGTGTAAAAGACTTACCAAATGCCGACAAGGAAACTGACCCATTCAAACTTTCTGGTGCTCAAGTTGTTGACAATTTAAAGCTTGCCTCTAACTTGGCCAATGTTGGTGATGCCAAGACCTTAGTCATTGCTCCATACTTCACTACCCACAAACAATTAAATGACAAAGAAAAGTTGGCATCTGGTGTTACCAAGGACTTAATTCGTGTCTCTGTTGGTATCGAATTTATTGATGACATTATTGCAGACTTCCAGCAATCTTTTGAAACTGTTTTCGCTGGCCAAAAACCATGACAGTTCGAGTTTATCATTATCAATACTGCCATTTCAAAGAATACGTAAATAATTAATAGTAGTGATTTTCCTAACTTTATTTAGTCAAAAAATTAGCCTTTTAATTCTGCTGTAACCCGTACATGCCCAAAATAGGGGGCGGGTTACACAGAATATATAACATCGTAGGTGTCTGGGTGAACAGTTTATTCCTGGCATCCACTAAATATAATGGAGCCCGCTTTTTAAGCTGGCATCCAGAAAAAAAAAGAATCCCAGCACCAAAATATTGTTTTCTTCACCAACCATCAGTTCATAGGTCCATTCTCTTAGCGCAACTACAGAGAACAGGGGCACAAACAGGCAAAAAACGGGCACAACCTCAATGGAGTGATGCAACCTGCCTGGAGTAAATGATGACACAAGGCAATTGACCCACGCATGTATCTATCTCATTTTCTTACACCTTCTATTACCTTCTGCTCTCTCTGATTTGGAAAAAGCTGAAAAAAAAGGTTGAAACCAGTTCCCTGAAATTATTCCCCTACTTGACTAATAAGTATATAAAGACGGTAGGTATTGATTGTAATTCTGTAAATCTATTTCTTAAACTTCTTAAATTCTACTTTTATAGTTAGTCTTTTTTTTAGTTTTAAAACACCAAGAACTTAGTTTCGAATAAACACACATAAACAAACAAAGGATCCAAAACAATGCCATCTGCTGAATCTATGACACCTTCATCTGCTTTGGGTCAATTAAAAGCTACAGGTCAACATGTTTTGTCTAAATTACAACAACAAACTTCTAATGCTGATATTATTGATATTAGGAGAGTTGCAGTTGAAATTAATTTAAAAACTGAAATTACTTCTATGTTTAGACCTAAAGATGGTCCAAGGCAATTACCAACTTTGTTGTTGTATAATGAAAGGGGTTTGCAATTGTTTGAAAGGATTACATATTTGGAAGAATATTATTTGACAAATGATGAAATTAAAATTTTAACTAAACATGCAACAGAAATGGCTTCTTTTATTCCATCAGGTGCTATGATTATTGAATTGGGTTCAGGTAATTTGAGGAAAGTTAATTTGTTGTTAGAAGCATTAGATAATGCAGGTAAAGCTATTGATTATTATGCTTTAGATTTGTCTAGGGAAGAATTGGAAAGGACTTTAGCACAAGTTCCATCTTATAAACATGTTAAATGTCATGGTTTGTTAGGTACTTACGATGATGGTAGAGATTGGTTAAAAGCACCTGAAAATATTAATAAACAAAAATGTATTTTGCATTTGGGTTCATCTATTGGTAATTTTAATAGATCAGATGCAGCTACATTTTTAAAAGGTTTTACAGATGTTTTGGGTCCAAATGATAAAATGTTGATTGGTGTTGATGCATGTAATGATCCAGCTAGAGTTTATCATGCTTATAATGATAAAGTTGGTATTACTCATGAATTTATTTTAAATGGTTTGAGGAATGCAAATGAAATTATTGGTGAAACTGCATTTATTGAAGGTGATTGGAGAGTTATTGGTGAATATGTTTATGATGAAGAAGGTGGTAGACATCAAGCATTTTATGCTCCAACTAGGGATACTATGGTTATGGGTGAATTGATTCGCTCACATGATAGGATTCAAATTGAACAATCTTTAAAATATTCTAAAGAAGAATCAGAAAGGTTGTGGTCTACAGCTGGTTTGGAACAAGTTTCTGAATGGACTTATGGTAATGAATATGGTTTACATTTGTTAGCTAAATCTAGGATGTCTTTTTCTTTGATTCCATCAGTTTATGCAAGGTCTGCATTGCCTACATTAGATGATTGGGAAGCATTGTGGGCTACTTGGGATGTTGTTACTAGGCAAATGTTACCACAAGAAGAATTGTTGGAAAAACCAATTAAATTGAGGAATGCTTGTATTTTTTATTTAGGTCATATTCCTACATTTTTGGATATTCAATTGACTAAAACTACTAAACAAGCTCCATCTGAACCAGCACATTTTTGTAAAATTTTTGAAAGGGGTATTGATCCAGATGTTGATAATCCAGAATTGTGTCATGCTCATTCAGAAATTCCTGATGAATGGCCTCCTGTTGAAGAAATTTTAACTTATCAAGAAACTGTTAGATCAAGGTTAAGGGGTTTGTATGCACATGGTATTGCTAATATTCCAAGGAATGTTGGTAGGGCTATTTGGGTTGGTTTTGAACATGAATTAATGCATATTGAAACTTTGTTGTATATGATGTTACAATCAGATAAAACTTTGATTCCAACTCATATTCCTAGACCAGATTTTGATAAATTAGCTAGGAAAGCAGAATCTGAAAGAGTTCCAAATCAATGGTTTAAAATTCCAGCACAAGAAATTACTATTGGTTTGGATGATCCTGAAGATGGTTCAGATATTAATAAACATTATGGTTGGGATAATGAAAAACCACCTAGGAGAGTTCAAGTTGCTGCATTTCAAGCTCAAGGTAGACCTATTACAAATGAAGAATATGCTCAATATTTGTTGGAAAAAAATATTGATAAATTGCCAGCTTCTTGGGCTAGGTTAGATAATGAAAATATTTCTAATGGTACTACTAATTCAGTTTCAGGTCATCATTCTAATAGGACTTCTAAACAACAATTGCCATCATCATTTTTAGAAAAAACTGCTGTTAGGACAGTTTATGGTTTAGTTCCTTTAAAACATGCTTTAGATTGGCCAGTTTTTGCTTCTTATGATGAATTAGCAGGTTGTGCTGCATATATGGGTGGTAGGATTCCTACATTTGAAGAAACTAGGTCTATTTATGCCTACGCTGATGCTTTAAAAAAAAAAAAAGAAGCAGAAAGGCAATTAGGTAGGACAGTTCCAGCAGTTAATGCACATTTAACTAATAATGGTGTTGAAATTACACCTCCTTCTTCACCTTCTTCAGAAACACCAGCAGAATCTTCATCACCATCTGATTCTAATACTACTTTGATTACTACAGAAGATTTGTTTTCAGATTTAGATGGTGCAAATGTTGGTTTTCATAATTGGCATCCTATGCCTATTACTTCTAAAGGTAATACTTTAGTTGGTCAAGGTGAATTGGGTGGTGTTTGGGAATGGACTTCTTCAGTTTTGAGGAAATGGGAAGGTTTTGAACCTATGGAATTGTATCCTGGTTATACAGCAGATTTTTTTGATGAAAAACATAATATTGTTTTAGGTGGTTCTTGGGCAACTCATCCTAGGATTGCAGGTAGGAAATCTTTTGTTAATTGGTATCAAAGGAATTATCCTTATGCATGGGTTGGTGCTAGAGTTGTTAGGGATTTATGAGGATCCGCGCAATGATTGAATAGTCAAAGATTTTTTTTTTTTAATTTTTTTTTTTTAATTTTTTTTTTTTTTCATAGAACTTTTTATTTAAATAAATCACGTCTATATATGTATCAGTATAACGTAAAAAAAAAAACACCGTCAGTTAAACAAAACATAAATAAAAAAAAAAAGAAGTGTCAAATCAAGTGTCAAATGGCTGGCAACTAATAGGGACACTACCAATATATTATCATATACGGTGTTAGACGATGACATAAGATACGAGGAACTGTCATCGAAGTTAGAGGAAGCTGAAATGCAAGGATTGATAATGTAATAGGATAATGAAACATATAAAACGGAATGAGGAATAATCGTAATATTAGTATATAGAGATAAAGATTCCATTTTGAGGATTCCTATATCCTCGAGGAGAACTTCTAGTATATTCTGTATACCTGATATTATAGCCT |
| donor DNA-2  (multi-copy integration at rDNA site for EGT biosynthesis, with constitutive expression of *EGT1* and *EGT2*) | TCTATGACGTCCTGTTCCAAGGAACATAGACAAGGAACGGCCCCAAAGTTGCCCTCTCCAAATTACAACTCGGGCACCGAAGGTACCAGATTTCAAATTTGAGCTTTTGCCGCTTCACTCGCCGTTACTAAGGCAATCCCGGTTGGTTTCTTTTCCTCCGCTTATTGATATGCTTAAGTTCAGCGGGTACTCCTACCTGATTTGAGGTCAAACTTTAAGAACATTGTTCGCCTAGACGCTCTCTTCTTATCGATAACGTTCCAATACGCTCAGTATAAAAAAAGATTAGCCGCAGTTGGTAAAACCTAAAACGACCGTACTTGCATTTTCAACATCGTATTTTCCGAAGCGTTGCATAATGAAAATGTGATAATAAGGCAGTGAGCTACTTTGTCACAACAAAGTACGAGAACAGATGAGGTTCCGTGGTGTTTTTGGGGCAACCGCACTATTTGGAGCGCTGTATATATTTATATCCTACTACAAAAAATCATAAATGACACTGAATTGGACACATGCTAGCGTCAAATACCTTGCCTGGTAAAGTTGTGTGCTAGTGTCTCCCGTCTTCTGTGAGCTCTTATTCAACTGGTTGTGGAACTAAATATTCCCTCCTCCTAATCCTTTCACACAAAGCCTTTAAAATATCACCAGCTGCTTCATAATCTTGTTCATCTAAATAAATTTGAGCTGATAACCTAACCCAATATCTATCTTGCATAACAAACAATGTCATAAAAGTTTTATAATCATCAAATAACCTTTCTCTCATCCATTTATCAACTAAAGCAACATTTTCCCTAGCAACAACAACATCAGGTGCAGCAATTGCAGCAGATGGTGCTGCATCCAATGCAGTAGAAGCATCTTCATCATCAACTCTCATAGGTAAAGCAACATTACCCATAGCACAATTAGTTAAAGTTTCTGTTTCATTATCCAAATGAGTAGTACCCAATGCCCTAGCAACAATCCTAATACCTTTTTTATTTAATGCCCACAAATACCTTAAAATATTTTCTTCACCACCACAAACTCTTTCCCTCCATGCAATTGCATCAGCAACACACAAATATGGACCATTATCTCTAGTACCAACAAATTCAAAATTAGCAACATAGCGGGATTTAGAACCAGGTGGTGCAGGAGCTGCTGAAGGTGGAACATAACCATGAGATGTAGCCAATGCTGTCCTTAATAAACATTGAGTCCTAGCTGGTGTATATAACATTGCACAACCCCTAGGAACTAATAACCATTTATGACAATTAGAAACAAAAAAATCAGGATCGGCTGCTGTTAAATCTAATCTAACCATACCAACACCTTGTGCACCATCAACTAAAGATAAAATACCCAATTCTCTACAAACTCTAACCGCTGCTTCCCAAGGAAAAACAACACCTGGCCTTGAAGTAACAACATCCATCATAGCCAATCTTGCCCTTTTACCTTCTCTAGCAACTTGTGTTGCTGCACCCCTCAAAGCTGCAATAACATCAGCATCTTCAACTGGATATTCCAATTCAATAGTCCTATGTTCAACTTTACCTGCAAAATATTCAACAATATAATCAGCAGCATTACCACATGCTTCATAAACTGTAGAAAAAGACAAAATAACATCTTTTTGACCACCTTTTTCCAAAGAATCCCACCTCAAATTCCTCAAAACTGTATTAACACCTTCAGTTGCATTACCAACAAAAACAACTGTATCTAATGGAGCATTAACAATTTTAGCAACCGCCGCTCGACTCCTATGTAATAATTTAGATTCTTCATACCTAATAAAATGATCAGGCCTAGCTTCTGCTTGATCTTGATATGCTCTTAATTTATCCCTAATATACAATGGATAAGTACCAAATGAACCATGATTTAAATTCCTCCATGCTGGATCAAATAAAAATTCTGATTTCCAAGCTCTACCAAATGCCAAAACTTCCCTTTGTGGAGTTTCTTGTAAAGGTTCTGATTCTGCTTCCAATTTATGTTCTGGTTCTTTCCTAGGACCCAATTGACCTTCAGTTTCTGGTTCACCATCAGGAGCCCTTTCAGGAACATGTTCCAATTTTAATTCTGGTTCTCTTTCTGGCCTAGGTTCACCACCTTGACCCCTACCCCTCAAAACTAATTCTTCACCTTCTAATAAACCCATTGTTTTGAGCTCTATTGATATAGTGTTTAAGCGAATGACAGAAGATTAATTTCTTGGTATGTTAGGAAAGAATAAAGGAGAATAAGAATAATTAGAACAATGTAGGATGGAAAGAAAGATTATCAAGCATGCCGACTTTATATACTTGAACGGAGGCAAAGGATGCAAAATTTTCTCACATTTCTTTCTGCCGTTATGTTGGAAGTAAGACTCCCATTATCGCAATACTGCAACACGAATATGCAAAATTTGCTGAGTTATCGCAGATAGTTGTTGCAAAGATAGCGGCGTAGGTGGCCGCGAAATGGGGAATTCCAAAACAAACGGTTTTTTTACTCCTGAGAAATACTTGTACGGGATAATCCAGGGCCTACCACCCACGCTTCGAGGATTGGCTTTTATTTTTTTTTTTTTGGTGGCGTTTTATTTCTTTCCCGCTTTCTGGGACTTGTGCGGAGTTTTGAGAGGGGCGCGCGGCAAAGGATTCCCAAAACGGAAATCAGACGCCAATAGCCAGCACTCAAAGCAGTTCTGGACCCATTCCGATTTTCCCATTTGGTTCTTGCGCGTGCTGATTCCGACACGCGCGTCTATAAATAGCATGAAGTATCCGCACACCGCAGCGTTAGTGAGGTGAGGGTGGCAGCAAGCTAATTCCCGCATCTGGAATCTGAACTGCCCCTTTTGGACTAACCGTGTGGTTCATGGGTGCCGCCATCTCTACTCACTCCATAATATTACATATAGATATAGGACAAGCCCGCATTTTCATACTGAAAGGTAAACTTCTATTATTATAGTGGTATCCAACGTTCACCGCTTCCAGCATAGCAGAAATTACGTGTTTTTGCATATGTTATGCTGATCATTGTATGCTTACTACCATTTTTCTTTGCTTCGCCTTGCCTTCTTTGACGTTTTTTTGAAGCAAAAAAAAAGTCAAGACAGATGTGCTTACAAAACCATGTAAGGCTCATTTTCAAAGAAGCTACTAATAGAAAGAGAACAAAGCGTTTACGAGTCTGGAAAATCAATGCCATCTCATTTCGATACTGTTCAACTACACGCCGGCCAAGAGAACCCTGGTGACAATGCTCACAGATCCAGAGCTGTACCAATTTACGCCACCACTTCTTATGTTTTCGAAAACTCTAAGCATGGTTCGCAATTGTTTGGTCTAGAAGTTCCAGGTTACGTCTATTCCCGTTTCCAAAACCCAACCAGTAATGTTTTGGAAGAAAGAATTGCTGCTTTAGAAGGTGGTGCTGCTGCTTTGGCTGTTTCCTCCGGTCAAGCCGCTCAAACCCTTGCCATCCAAGGTTTGGCACACACTGGTGACAACATCGTTTCCACTTCTTACTTATACGGTGGTACTTATAACCAGTTCAAAATCTCGTTCAAAAGATTTGGTATCGAGGCTAGATTTGTTGAAGGTGACAATCCAGAAGAATTCGAAAAGGTCTTTGATGAAAGAACCAAGGCTGTTTATTTGGAAACCATTGGTAATCCAAAGTACAATGTTCCGGATTTTGAAAAAATTGTTGCAATTGCTCACAAACACGGTATTCCAGTTGTCGTTGACAACACATTTGGTGCCGGTGGTTACTTCTGTCAGCCAATTAAATACGGTGCTGATATTGTAACACATTCTGCTACCAAATGGATTGGTGGTCATGGTACTACTATCGGTGGTATTATTGTTGACTCTGGTAAGTTCCCATGGAAGGACTACCCAGAAAAGTTCCCTCAATTCTCTCAACCTGCCGAAGGATATCACGGTACTATCTACAATGAAGCCTACGGTAACTTGGCATACATCGTTCATGTTAGAACTGAACTATTAAGAGATTTGGGTCCATTGATGAACCCATTTGCCTCTTTCTTGCTACTACAAGGTGTTGAAACATTATCTTTGAGAGCTGAAAGACACGGTGAAAATGCATTGAAGTTAGCCAAATGGTTAGAACAATCCCCATACGTATCTTGGGTTTCATACCCTGGTTTAGCATCTCATTCTCATCATGAAAATGCTAAGAAGTATCTATCTAACGGTTTCGGTGGTGTCTTATCTTTCGGTGTAAAAGACTTACCAAATGCCGACAAGGAAACTGACCCATTCAAACTTTCTGGTGCTCAAGTTGTTGACAATTTAAAGCTTGCCTCTAACTTGGCCAATGTTGGTGATGCCAAGACCTTAGTCATTGCTCCATACTTCACTACCCACAAACAATTAAATGACAAAGAAAAGTTGGCATCTGGTGTTACCAAGGACTTAATTCGTGTCTCTGTTGGTATCGAATTTATTGATGACATTATTGCAGACTTCCAGCAATCTTTTGAAACTGTTTTCGCTGGCCAAAAACCATGACAGTTCGAGTTTATCATTATCAATACTGCCATTTCAAAGAATACGTAAATAATTAATAGTAGTGATTTTCCTAACTTTATTTAGTCAAAAAATTAGCCTTTTAATTCTGCTGTAACCCGTACATGCCCAAAATAGGGGGCGGGTTACACAGAATATATAACATCGTAGGTGTCTGGGTGAACAGTTTATTCCTGGCATCCACTAAATATAATGGAGCCCGCTTTTTAAGCTGGCATCCAGAAAAAAAAAGAATCCCAGCACCAAAATATTGTTTTCTTCACCAACCATCAGTTCATAGGTCCATTCTCTTAGCGCAACTACAGAGAACAGGGGCACAAACAGGCAAAAAACGGGCACAACCTCAATGGAGTGATGCAACCTGCCTGGAGTAAATGATGACACAAGGCAATTGACCCACGCATGTATCTATCTCATTTTCTTACACCTTCTATTACCTTCTGCTCTCTCTGATTTGGAAAAAGCTGAAAAAAAAGGTTGAAACCAGTTCCCTGAAATTATTCCCCTACTTGACTAATAAGTATATAAAGACGGTAGGTATTGATTGTAATTCTGTAAATCTATTTCTTAAACTTCTTAAATTCTACTTTTATAGTTAGTCTTTTTTTTAGTTTTAAAACACCAAGAACTTAGTTTCGAATAAACACACATAAACAAACAAAGGATCCAAAACAATGCCATCTGCTGAATCTATGACACCTTCATCTGCTTTGGGTCAATTAAAAGCTACAGGTCAACATGTTTTGTCTAAATTACAACAACAAACTTCTAATGCTGATATTATTGATATTAGGAGAGTTGCAGTTGAAATTAATTTAAAAACTGAAATTACTTCTATGTTTAGACCTAAAGATGGTCCAAGGCAATTACCAACTTTGTTGTTGTATAATGAAAGGGGTTTGCAATTGTTTGAAAGGATTACATATTTGGAAGAATATTATTTGACAAATGATGAAATTAAAATTTTAACTAAACATGCAACAGAAATGGCTTCTTTTATTCCATCAGGTGCTATGATTATTGAATTGGGTTCAGGTAATTTGAGGAAAGTTAATTTGTTGTTAGAAGCATTAGATAATGCAGGTAAAGCTATTGATTATTATGCTTTAGATTTGTCTAGGGAAGAATTGGAAAGGACTTTAGCACAAGTTCCATCTTATAAACATGTTAAATGTCATGGTTTGTTAGGTACTTACGATGATGGTAGAGATTGGTTAAAAGCACCTGAAAATATTAATAAACAAAAATGTATTTTGCATTTGGGTTCATCTATTGGTAATTTTAATAGATCAGATGCAGCTACATTTTTAAAAGGTTTTACAGATGTTTTGGGTCCAAATGATAAAATGTTGATTGGTGTTGATGCATGTAATGATCCAGCTAGAGTTTATCATGCTTATAATGATAAAGTTGGTATTACTCATGAATTTATTTTAAATGGTTTGAGGAATGCAAATGAAATTATTGGTGAAACTGCATTTATTGAAGGTGATTGGAGAGTTATTGGTGAATATGTTTATGATGAAGAAGGTGGTAGACATCAAGCATTTTATGCTCCAACTAGGGATACTATGGTTATGGGTGAATTGATTCGCTCACATGATAGGATTCAAATTGAACAATCTTTAAAATATTCTAAAGAAGAATCAGAAAGGTTGTGGTCTACAGCTGGTTTGGAACAAGTTTCTGAATGGACTTATGGTAATGAATATGGTTTACATTTGTTAGCTAAATCTAGGATGTCTTTTTCTTTGATTCCATCAGTTTATGCAAGGTCTGCATTGCCTACATTAGATGATTGGGAAGCATTGTGGGCTACTTGGGATGTTGTTACTAGGCAAATGTTACCACAAGAAGAATTGTTGGAAAAACCAATTAAATTGAGGAATGCTTGTATTTTTTATTTAGGTCATATTCCTACATTTTTGGATATTCAATTGACTAAAACTACTAAACAAGCTCCATCTGAACCAGCACATTTTTGTAAAATTTTTGAAAGGGGTATTGATCCAGATGTTGATAATCCAGAATTGTGTCATGCTCATTCAGAAATTCCTGATGAATGGCCTCCTGTTGAAGAAATTTTAACTTATCAAGAAACTGTTAGATCAAGGTTAAGGGGTTTGTATGCACATGGTATTGCTAATATTCCAAGGAATGTTGGTAGGGCTATTTGGGTTGGTTTTGAACATGAATTAATGCATATTGAAACTTTGTTGTATATGATGTTACAATCAGATAAAACTTTGATTCCAACTCATATTCCTAGACCAGATTTTGATAAATTAGCTAGGAAAGCAGAATCTGAAAGAGTTCCAAATCAATGGTTTAAAATTCCAGCACAAGAAATTACTATTGGTTTGGATGATCCTGAAGATGGTTCAGATATTAATAAACATTATGGTTGGGATAATGAAAAACCACCTAGGAGAGTTCAAGTTGCTGCATTTCAAGCTCAAGGTAGACCTATTACAAATGAAGAATATGCTCAATATTTGTTGGAAAAAAATATTGATAAATTGCCAGCTTCTTGGGCTAGGTTAGATAATGAAAATATTTCTAATGGTACTACTAATTCAGTTTCAGGTCATCATTCTAATAGGACTTCTAAACAACAATTGCCATCATCATTTTTAGAAAAAACTGCTGTTAGGACAGTTTATGGTTTAGTTCCTTTAAAACATGCTTTAGATTGGCCAGTTTTTGCTTCTTATGATGAATTAGCAGGTTGTGCTGCATATATGGGTGGTAGGATTCCTACATTTGAAGAAACTAGGTCTATTTATGCCTACGCTGATGCTTTAAAAAAAAAAAAAGAAGCAGAAAGGCAATTAGGTAGGACAGTTCCAGCAGTTAATGCACATTTAACTAATAATGGTGTTGAAATTACACCTCCTTCTTCACCTTCTTCAGAAACACCAGCAGAATCTTCATCACCATCTGATTCTAATACTACTTTGATTACTACAGAAGATTTGTTTTCAGATTTAGATGGTGCAAATGTTGGTTTTCATAATTGGCATCCTATGCCTATTACTTCTAAAGGTAATACTTTAGTTGGTCAAGGTGAATTGGGTGGTGTTTGGGAATGGACTTCTTCAGTTTTGAGGAAATGGGAAGGTTTTGAACCTATGGAATTGTATCCTGGTTATACAGCAGATTTTTTTGATGAAAAACATAATATTGTTTTAGGTGGTTCTTGGGCAACTCATCCTAGGATTGCAGGTAGGAAATCTTTTGTTAATTGGTATCAAAGGAATTATCCTTATGCATGGGTTGGTGCTAGAGTTGTTAGGGATTTATGAGGATCCGCGCAATGATTGAATAGTCAAAGATTTTTTTTTTTTAATTTTTTTTTTTTAATTTTTTTTTTTTTTCATAGAACTTTTTATTTAAATAAATCACGTCTATATATGTATCAGTATAACGTAAAAAAAAAAACACCGTCAGTTAAACAAAACATAAATAAAAAAAAAAAGAAGTGTCAAATCAAGTGTCAAATATTATACCTCAAGCACGCAGAGAAACCTCTCTTTGGAAAAAAAACATCCAATGAAAAGGCCAGCAATTTCAAGTTAACTCCAAAGAGTATCACTCACTACCAAACAGAATGTTTGAGAAGGAAATGACGCTCAAACAGGCATGCCCCCTGGAATACCAAGGGGCGCAATGTGCGTTCAAAGATTCGATGATTCACGGAATTCTGCAATTCACATTACGTATCGCATTTCGCTGCGTTCTTCATCGATGCGAGAACCAAGAGATCCGTTGTTGAAAGTTTTTAATATTTTAAAATTTCCAGTTACGAAAATTCTTGTTTTTGACAAAAATTTAATGAATAGATAAAATTGTTTGTGTTTGTTACCTCTGGGCC |
| donor DNA-3  (multi-copy integration at δ site for EGT biosynthesis, with inducible expression of *EGT1* and *EGT2*) | TTGTTGGAATAAAAATCAACTATCATCTACTAACTAGTATTTACGTTACTAGTATATTATCATATACGGTGTTAGAAGATGACGCAAATGATGAGAAATAGTCATCTAAATTAGTGGAAGCTGAAACGCAAGGATTGATAATGTAATAGGATCAATGAATATTAACATATAAAATGATGATAATAATATTTATAGAATTGTGTAGAATTGCAGATTCCCTTTTATTTTCAACATCGTATTTTCCGAAGCGTTGCATAATGAAAATGTGATAATAAGGCAGTGAGCTACTTTGTCACAACAAAGTACGAGAACAGATGAGGTTCCGTGGTGTTTTTGGGGCAACCGCACTATTTGGAGCGCTGTATATATTTATATCCTACTACAAAAAATCATAAATGACACTGAATTGGACACATGCTAGCGTCAAATACCTTGCCTGGTAAAGTTGTGTGCTAGTGTCTCCCGTCTTCTGTGAGCTCTTATTCAACTGGTTGTGGAACTAAATATTCCCTCCTCCTAATCCTTTCACACAAAGCCTTTAAAATATCACCAGCTGCTTCATAATCTTGTTCATCTAAATAAATTTGAGCTGATAACCTAACCCAATATCTATCTTGCATAACAAACAATGTCATAAAAGTTTTATAATCATCAAATAACCTTTCTCTCATCCATTTATCAACTAAAGCAACATTTTCCCTAGCAACAACAACATCAGGTGCAGCAATTGCAGCAGATGGTGCTGCATCCAATGCAGTAGAAGCATCTTCATCATCAACTCTCATAGGTAAAGCAACATTACCCATAGCACAATTAGTTAAAGTTTCTGTTTCATTATCCAAATGAGTAGTACCCAATGCCCTAGCAACAATCCTAATACCTTTTTTATTTAATGCCCACAAATACCTTAAAATATTTTCTTCACCACCACAAACTCTTTCCCTCCATGCAATTGCATCAGCAACACACAAATATGGACCATTATCTCTAGTACCAACAAATTCAAAATTAGCAACATAGCGGGATTTAGAACCAGGTGGTGCAGGAGCTGCTGAAGGTGGAACATAACCATGAGATGTAGCCAATGCTGTCCTTAATAAACATTGAGTCCTAGCTGGTGTATATAACATTGCACAACCCCTAGGAACTAATAACCATTTATGACAATTAGAAACAAAAAAATCAGGATCGGCTGCTGTTAAATCTAATCTAACCATACCAACACCTTGTGCACCATCAACTAAAGATAAAATACCCAATTCTCTACAAACTCTAACCGCTGCTTCCCAAGGAAAAACAACACCTGGCCTTGAAGTAACAACATCCATCATAGCCAATCTTGCCCTTTTACCTTCTCTAGCAACTTGTGTTGCTGCACCCCTCAAAGCTGCAATAACATCAGCATCTTCAACTGGATATTCCAATTCAATAGTCCTATGTTCAACTTTACCTGCAAAATATTCAACAATATAATCAGCAGCATTACCACATGCTTCATAAACTGTAGAAAAAGACAAAATAACATCTTTTTGACCACCTTTTTCCAAAGAATCCCACCTCAAATTCCTCAAAACTGTATTAACACCTTCAGTTGCATTACCAACAAAAACAACTGTATCTAATGGAGCATTAACAATTTTAGCAACCGCCGCTCGACTCCTATGTAATAATTTAGATTCTTCATACCTAATAAAATGATCAGGCCTAGCTTCTGCTTGATCTTGATATGCTCTTAATTTATCCCTAATATACAATGGATAAGTACCAAATGAACCATGATTTAAATTCCTCCATGCTGGATCAAATAAAAATTCTGATTTCCAAGCTCTACCAAATGCCAAAACTTCCCTTTGTGGAGTTTCTTGTAAAGGTTCTGATTCTGCTTCCAATTTATGTTCTGGTTCTTTCCTAGGACCCAATTGACCTTCAGTTTCTGGTTCACCATCAGGAGCCCTTTCAGGAACATGTTCCAATTTTAATTCTGGTTCTCTTTCTGGCCTAGGTTCACCACCTTGACCCCTACCCCTCAAAACTAATTCTTCACCTTCTAATAAACCCATTTTCAAAAATTCTTACTTTTTTTTTGGATGGACGCAAAGAAGTTTAATAATCATATTACATGGCATTACCACCATATACATATCCATATACATATCCATATCTAATCTTACTTATATGTTGTGGAAATGTAAAGAGCCCCATTATCTTAGCCTAAAAAAACCTTCTCTTTGGAACTTTCAGTAATACGCTTAACTGCTCATTGCTATATTGAAGTACGGATTAGAAGCCGCCGAGCGGGTGACAGCCCTCCGAAGGAAGACTCTCCTCCGTGCGTCCTCGTCTTCACCGGTCGCGTTCCTGAAACGCAGATGTGCCTCGCGCCGCACTGCTCCGAACAATAAAGATTCTACAATACTAGCTTTTATGGTTATGAAGAGGAAAAATTGGCAGTAACCTGGCCCCACAAACCTTCAAATGAACGAATCAAATTAACAACCATAGGATGATAATGCGATTAGTTTTTTAGCCTTATTTCTGGGGTAATTAATCAGCGAAGCGATGATTTTTGATCTATTAACAGATATATAAATGCAAAAACTGCATAACCACTTTAACTAATACTTTCAACATTTTCGGTTTGTATTACTTCTTATTCAAATGTAATAAAAGTATCAACAAAAAATTGTTAATATACCTCTATACTTTAACGTCAAGGAGAAAAAACATGCCATCTGCTGAATCTATGACACCTTCATCTGCTTTGGGTCAATTAAAAGCTACAGGTCAACATGTTTTGTCTAAATTACAACAACAAACTTCTAATGCTGATATTATTGATATTAGGAGAGTTGCAGTTGAAATTAATTTAAAAACTGAAATTACTTCTATGTTTAGACCTAAAGATGGTCCAAGGCAATTACCAACTTTGTTGTTGTATAATGAAAGGGGTTTGCAATTGTTTGAAAGGATTACATATTTGGAAGAATATTATTTGACAAATGATGAAATTAAAATTTTAACTAAACATGCAACAGAAATGGCTTCTTTTATTCCATCAGGTGCTATGATTATTGAATTGGGTTCAGGTAATTTGAGGAAAGTTAATTTGTTGTTAGAAGCATTAGATAATGCAGGTAAAGCTATTGATTATTATGCTTTAGATTTGTCTAGGGAAGAATTGGAAAGGACTTTAGCACAAGTTCCATCTTATAAACATGTTAAATGTCATGGTTTGTTAGGTACTTACGATGATGGTAGAGATTGGTTAAAAGCACCTGAAAATATTAATAAACAAAAATGTATTTTGCATTTGGGTTCATCTATTGGTAATTTTAATAGATCAGATGCAGCTACATTTTTAAAAGGTTTTACAGATGTTTTGGGTCCAAATGATAAAATGTTGATTGGTGTTGATGCATGTAATGATCCAGCTAGAGTTTATCATGCTTATAATGATAAAGTTGGTATTACTCATGAATTTATTTTAAATGGTTTGAGGAATGCAAATGAAATTATTGGTGAAACTGCATTTATTGAAGGTGATTGGAGAGTTATTGGTGAATATGTTTATGATGAAGAAGGTGGTAGACATCAAGCATTTTATGCTCCAACTAGGGATACTATGGTTATGGGTGAATTGATTCGCTCACATGATAGGATTCAAATTGAACAATCTTTAAAATATTCTAAAGAAGAATCAGAAAGGTTGTGGTCTACAGCTGGTTTGGAACAAGTTTCTGAATGGACTTATGGTAATGAATATGGTTTACATTTGTTAGCTAAATCTAGGATGTCTTTTTCTTTGATTCCATCAGTTTATGCAAGGTCTGCATTGCCTACATTAGATGATTGGGAAGCATTGTGGGCTACTTGGGATGTTGTTACTAGGCAAATGTTACCACAAGAAGAATTGTTGGAAAAACCAATTAAATTGAGGAATGCTTGTATTTTTTATTTAGGTCATATTCCTACATTTTTGGATATTCAATTGACTAAAACTACTAAACAAGCTCCATCTGAACCAGCACATTTTTGTAAAATTTTTGAAAGGGGTATTGATCCAGATGTTGATAATCCAGAATTGTGTCATGCTCATTCAGAAATTCCTGATGAATGGCCTCCTGTTGAAGAAATTTTAACTTATCAAGAAACTGTTAGATCAAGGTTAAGGGGTTTGTATGCACATGGTATTGCTAATATTCCAAGGAATGTTGGTAGGGCTATTTGGGTTGGTTTTGAACATGAATTAATGCATATTGAAACTTTGTTGTATATGATGTTACAATCAGATAAAACTTTGATTCCAACTCATATTCCTAGACCAGATTTTGATAAATTAGCTAGGAAAGCAGAATCTGAAAGAGTTCCAAATCAATGGTTTAAAATTCCAGCACAAGAAATTACTATTGGTTTGGATGATCCTGAAGATGGTTCAGATATTAATAAACATTATGGTTGGGATAATGAAAAACCACCTAGGAGAGTTCAAGTTGCTGCATTTCAAGCTCAAGGTAGACCTATTACAAATGAAGAATATGCTCAATATTTGTTGGAAAAAAATATTGATAAATTGCCAGCTTCTTGGGCTAGGTTAGATAATGAAAATATTTCTAATGGTACTACTAATTCAGTTTCAGGTCATCATTCTAATAGGACTTCTAAACAACAATTGCCATCATCATTTTTAGAAAAAACTGCTGTTAGGACAGTTTATGGTTTAGTTCCTTTAAAACATGCTTTAGATTGGCCAGTTTTTGCTTCTTATGATGAATTAGCAGGTTGTGCTGCATATATGGGTGGTAGGATTCCTACATTTGAAGAAACTAGGTCTATTTATGCCTACGCTGATGCTTTAAAAAAAAAAAAAGAAGCAGAAAGGCAATTAGGTAGGACAGTTCCAGCAGTTAATGCACATTTAACTAATAATGGTGTTGAAATTACACCTCCTTCTTCACCTTCTTCAGAAACACCAGCAGAATCTTCATCACCATCTGATTCTAATACTACTTTGATTACTACAGAAGATTTGTTTTCAGATTTAGATGGTGCAAATGTTGGTTTTCATAATTGGCATCCTATGCCTATTACTTCTAAAGGTAATACTTTAGTTGGTCAAGGTGAATTGGGTGGTGTTTGGGAATGGACTTCTTCAGTTTTGAGGAAATGGGAAGGTTTTGAACCTATGGAATTGTATCCTGGTTATACAGCAGATTTTTTTGATGAAAAACATAATATTGTTTTAGGTGGTTCTTGGGCAACTCATCCTAGGATTGCAGGTAGGAAATCTTTTGTTAATTGGTATCAAAGGAATTATCCTTATGCATGGGTTGGTGCTAGAGTTGTTAGGGATTTATGAGGATCCGCGCAATGATTGAATAGTCAAAGATTTTTTTTTTTTAATTTTTTTTTTTTAATTTTTTTTTTTTTTCATAGAACTTTTTATTTAAATAAATCACGTCTATATATGTATCAGTATAACGTAAAAAAAAAAACACCGTCAGTTAAACAAAACATAAATAAAAAAAAAAAGAAGTGTCAAATCAAGTGTCAAATCCGCCATCTCTACTCACTCCATAATATTACATATAGATATAGGACAAGCCCGCATTTTCATACTGAAAGGTAAACTTCTATTATTATAGTGGTATCCAACGTTCACCGCTTCCAGCATAGCAGAAATTACGTGTTTTTGCATATGTTATGCTGATCATTGTATGCTTACTACCATTTTTCTTTGCTTCGCCTTGCCTTCTTTGACGTTTTTTTGAAGCAAAAAAAAAGTCAAGACAGATGTGCTTACAAAACCATGTAAGGCTCATTTTCAAAGAAGCTACTAATAGAAAGAGAACAAAGCGTTTACGAGTCTGGAAAATCAATGCCATCTCATTTCGATACTGTTCAACTACACGCCGGCCAAGAGAACCCTGGTGACAATGCTCACAGATCCAGAGCTGTACCAATTTACGCCACCACTTCTTATGTTTTCGAAAACTCTAAGCATGGTTCGCAATTGTTTGGTCTAGAAGTTCCAGGTTACGTCTATTCCCGTTTCCAAAACCCAACCAGTAATGTTTTGGAAGAAAGAATTGCTGCTTTAGAAGGTGGTGCTGCTGCTTTGGCTGTTTCCTCCGGTCAAGCCGCTCAAACCCTTGCCATCCAAGGTTTGGCACACACTGGTGACAACATCGTTTCCACTTCTTACTTATACGGTGGTACTTATAACCAGTTCAAAATCTCGTTCAAAAGATTTGGTATCGAGGCTAGATTTGTTGAAGGTGACAATCCAGAAGAATTCGAAAAGGTCTTTGATGAAAGAACCAAGGCTGTTTATTTGGAAACCATTGGTAATCCAAAGTACAATGTTCCGGATTTTGAAAAAATTGTTGCAATTGCTCACAAACACGGTATTCCAGTTGTCGTTGACAACACATTTGGTGCCGGTGGTTACTTCTGTCAGCCAATTAAATACGGTGCTGATATTGTAACACATTCTGCTACCAAATGGATTGGTGGTCATGGTACTACTATCGGTGGTATTATTGTTGACTCTGGTAAGTTCCCATGGAAGGACTACCCAGAAAAGTTCCCTCAATTCTCTCAACCTGCCGAAGGATATCACGGTACTATCTACAATGAAGCCTACGGTAACTTGGCATACATCGTTCATGTTAGAACTGAACTATTAAGAGATTTGGGTCCATTGATGAACCCATTTGCCTCTTTCTTGCTACTACAAGGTGTTGAAACATTATCTTTGAGAGCTGAAAGACACGGTGAAAATGCATTGAAGTTAGCCAAATGGTTAGAACAATCCCCATACGTATCTTGGGTTTCATACCCTGGTTTAGCATCTCATTCTCATCATGAAAATGCTAAGAAGTATCTATCTAACGGTTTCGGTGGTGTCTTATCTTTCGGTGTAAAAGACTTACCAAATGCCGACAAGGAAACTGACCCATTCAAACTTTCTGGTGCTCAAGTTGTTGACAATTTAAAGCTTGCCTCTAACTTGGCCAATGTTGGTGATGCCAAGACCTTAGTCATTGCTCCATACTTCACTACCCACAAACAATTAAATGACAAAGAAAAGTTGGCATCTGGTGTTACCAAGGACTTAATTCGTGTCTCTGTTGGTATCGAATTTATTGATGACATTATTGCAGACTTCCAGCAATCTTTTGAAACTGTTTTCGCTGGCCAAAAACCATGAGGCTGGCAACTAATAGGGACACTACCAATATATTATCATATACGGTGTTAGACGATGACATAAGATACGAGGAACTGTCATCGAAGTTAGAGGAAGCTGAAATGCAAGGATTGATAATGTAATAGGATAATGAAACATATAAAACGGAATGAGGAATAATCGTAATATTAGTATATAGAGATAAAGATTCCATTTTGAGGATTCCTATATCCTCGAGGAGAACTTCTAGTATATTCTGTATACCTGATATTATAGCCT |
| donor DNA-4  (multi-copy integration at rDNA site for EGT biosynthesis, with inducible expression of *EGT1* and *EGT2*) | TCTATGACGTCCTGTTCCAAGGAACATAGACAAGGAACGGCCCCAAAGTTGCCCTCTCCAAATTACAACTCGGGCACCGAAGGTACCAGATTTCAAATTTGAGCTTTTGCCGCTTCACTCGCCGTTACTAAGGCAATCCCGGTTGGTTTCTTTTCCTCCGCTTATTGATATGCTTAAGTTCAGCGGGTACTCCTACCTGATTTGAGGTCAAACTTTAAGAACATTGTTCGCCTAGACGCTCTCTTCTTATCGATAACGTTCCAATACGCTCAGTATAAAAAAAGATTAGCCGCAGTTGGTAAAACCTAAAACGACCGTACTTGCATTTTCAACATCGTATTTTCCGAAGCGTTGCATAATGAAAATGTGATAATAAGGCAGTGAGCTACTTTGTCACAACAAAGTACGAGAACAGATGAGGTTCCGTGGTGTTTTTGGGGCAACCGCACTATTTGGAGCGCTGTATATATTTATATCCTACTACAAAAAATCATAAATGACACTGAATTGGACACATGCTAGCGTCAAATACCTTGCCTGGTAAAGTTGTGTGCTAGTGTCTCCCGTCTTCTGTGAGCTCTTATTCAACTGGTTGTGGAACTAAATATTCCCTCCTCCTAATCCTTTCACACAAAGCCTTTAAAATATCACCAGCTGCTTCATAATCTTGTTCATCTAAATAAATTTGAGCTGATAACCTAACCCAATATCTATCTTGCATAACAAACAATGTCATAAAAGTTTTATAATCATCAAATAACCTTTCTCTCATCCATTTATCAACTAAAGCAACATTTTCCCTAGCAACAACAACATCAGGTGCAGCAATTGCAGCAGATGGTGCTGCATCCAATGCAGTAGAAGCATCTTCATCATCAACTCTCATAGGTAAAGCAACATTACCCATAGCACAATTAGTTAAAGTTTCTGTTTCATTATCCAAATGAGTAGTACCCAATGCCCTAGCAACAATCCTAATACCTTTTTTATTTAATGCCCACAAATACCTTAAAATATTTTCTTCACCACCACAAACTCTTTCCCTCCATGCAATTGCATCAGCAACACACAAATATGGACCATTATCTCTAGTACCAACAAATTCAAAATTAGCAACATAGCGGGATTTAGAACCAGGTGGTGCAGGAGCTGCTGAAGGTGGAACATAACCATGAGATGTAGCCAATGCTGTCCTTAATAAACATTGAGTCCTAGCTGGTGTATATAACATTGCACAACCCCTAGGAACTAATAACCATTTATGACAATTAGAAACAAAAAAATCAGGATCGGCTGCTGTTAAATCTAATCTAACCATACCAACACCTTGTGCACCATCAACTAAAGATAAAATACCCAATTCTCTACAAACTCTAACCGCTGCTTCCCAAGGAAAAACAACACCTGGCCTTGAAGTAACAACATCCATCATAGCCAATCTTGCCCTTTTACCTTCTCTAGCAACTTGTGTTGCTGCACCCCTCAAAGCTGCAATAACATCAGCATCTTCAACTGGATATTCCAATTCAATAGTCCTATGTTCAACTTTACCTGCAAAATATTCAACAATATAATCAGCAGCATTACCACATGCTTCATAAACTGTAGAAAAAGACAAAATAACATCTTTTTGACCACCTTTTTCCAAAGAATCCCACCTCAAATTCCTCAAAACTGTATTAACACCTTCAGTTGCATTACCAACAAAAACAACTGTATCTAATGGAGCATTAACAATTTTAGCAACCGCCGCTCGACTCCTATGTAATAATTTAGATTCTTCATACCTAATAAAATGATCAGGCCTAGCTTCTGCTTGATCTTGATATGCTCTTAATTTATCCCTAATATACAATGGATAAGTACCAAATGAACCATGATTTAAATTCCTCCATGCTGGATCAAATAAAAATTCTGATTTCCAAGCTCTACCAAATGCCAAAACTTCCCTTTGTGGAGTTTCTTGTAAAGGTTCTGATTCTGCTTCCAATTTATGTTCTGGTTCTTTCCTAGGACCCAATTGACCTTCAGTTTCTGGTTCACCATCAGGAGCCCTTTCAGGAACATGTTCCAATTTTAATTCTGGTTCTCTTTCTGGCCTAGGTTCACCACCTTGACCCCTACCCCTCAAAACTAATTCTTCACCTTCTAATAAACCCATTTTCAAAAATTCTTACTTTTTTTTTGGATGGACGCAAAGAAGTTTAATAATCATATTACATGGCATTACCACCATATACATATCCATATACATATCCATATCTAATCTTACTTATATGTTGTGGAAATGTAAAGAGCCCCATTATCTTAGCCTAAAAAAACCTTCTCTTTGGAACTTTCAGTAATACGCTTAACTGCTCATTGCTATATTGAAGTACGGATTAGAAGCCGCCGAGCGGGTGACAGCCCTCCGAAGGAAGACTCTCCTCCGTGCGTCCTCGTCTTCACCGGTCGCGTTCCTGAAACGCAGATGTGCCTCGCGCCGCACTGCTCCGAACAATAAAGATTCTACAATACTAGCTTTTATGGTTATGAAGAGGAAAAATTGGCAGTAACCTGGCCCCACAAACCTTCAAATGAACGAATCAAATTAACAACCATAGGATGATAATGCGATTAGTTTTTTAGCCTTATTTCTGGGGTAATTAATCAGCGAAGCGATGATTTTTGATCTATTAACAGATATATAAATGCAAAAACTGCATAACCACTTTAACTAATACTTTCAACATTTTCGGTTTGTATTACTTCTTATTCAAATGTAATAAAAGTATCAACAAAAAATTGTTAATATACCTCTATACTTTAACGTCAAGGAGAAAAAACATGCCATCTGCTGAATCTATGACACCTTCATCTGCTTTGGGTCAATTAAAAGCTACAGGTCAACATGTTTTGTCTAAATTACAACAACAAACTTCTAATGCTGATATTATTGATATTAGGAGAGTTGCAGTTGAAATTAATTTAAAAACTGAAATTACTTCTATGTTTAGACCTAAAGATGGTCCAAGGCAATTACCAACTTTGTTGTTGTATAATGAAAGGGGTTTGCAATTGTTTGAAAGGATTACATATTTGGAAGAATATTATTTGACAAATGATGAAATTAAAATTTTAACTAAACATGCAACAGAAATGGCTTCTTTTATTCCATCAGGTGCTATGATTATTGAATTGGGTTCAGGTAATTTGAGGAAAGTTAATTTGTTGTTAGAAGCATTAGATAATGCAGGTAAAGCTATTGATTATTATGCTTTAGATTTGTCTAGGGAAGAATTGGAAAGGACTTTAGCACAAGTTCCATCTTATAAACATGTTAAATGTCATGGTTTGTTAGGTACTTACGATGATGGTAGAGATTGGTTAAAAGCACCTGAAAATATTAATAAACAAAAATGTATTTTGCATTTGGGTTCATCTATTGGTAATTTTAATAGATCAGATGCAGCTACATTTTTAAAAGGTTTTACAGATGTTTTGGGTCCAAATGATAAAATGTTGATTGGTGTTGATGCATGTAATGATCCAGCTAGAGTTTATCATGCTTATAATGATAAAGTTGGTATTACTCATGAATTTATTTTAAATGGTTTGAGGAATGCAAATGAAATTATTGGTGAAACTGCATTTATTGAAGGTGATTGGAGAGTTATTGGTGAATATGTTTATGATGAAGAAGGTGGTAGACATCAAGCATTTTATGCTCCAACTAGGGATACTATGGTTATGGGTGAATTGATTCGCTCACATGATAGGATTCAAATTGAACAATCTTTAAAATATTCTAAAGAAGAATCAGAAAGGTTGTGGTCTACAGCTGGTTTGGAACAAGTTTCTGAATGGACTTATGGTAATGAATATGGTTTACATTTGTTAGCTAAATCTAGGATGTCTTTTTCTTTGATTCCATCAGTTTATGCAAGGTCTGCATTGCCTACATTAGATGATTGGGAAGCATTGTGGGCTACTTGGGATGTTGTTACTAGGCAAATGTTACCACAAGAAGAATTGTTGGAAAAACCAATTAAATTGAGGAATGCTTGTATTTTTTATTTAGGTCATATTCCTACATTTTTGGATATTCAATTGACTAAAACTACTAAACAAGCTCCATCTGAACCAGCACATTTTTGTAAAATTTTTGAAAGGGGTATTGATCCAGATGTTGATAATCCAGAATTGTGTCATGCTCATTCAGAAATTCCTGATGAATGGCCTCCTGTTGAAGAAATTTTAACTTATCAAGAAACTGTTAGATCAAGGTTAAGGGGTTTGTATGCACATGGTATTGCTAATATTCCAAGGAATGTTGGTAGGGCTATTTGGGTTGGTTTTGAACATGAATTAATGCATATTGAAACTTTGTTGTATATGATGTTACAATCAGATAAAACTTTGATTCCAACTCATATTCCTAGACCAGATTTTGATAAATTAGCTAGGAAAGCAGAATCTGAAAGAGTTCCAAATCAATGGTTTAAAATTCCAGCACAAGAAATTACTATTGGTTTGGATGATCCTGAAGATGGTTCAGATATTAATAAACATTATGGTTGGGATAATGAAAAACCACCTAGGAGAGTTCAAGTTGCTGCATTTCAAGCTCAAGGTAGACCTATTACAAATGAAGAATATGCTCAATATTTGTTGGAAAAAAATATTGATAAATTGCCAGCTTCTTGGGCTAGGTTAGATAATGAAAATATTTCTAATGGTACTACTAATTCAGTTTCAGGTCATCATTCTAATAGGACTTCTAAACAACAATTGCCATCATCATTTTTAGAAAAAACTGCTGTTAGGACAGTTTATGGTTTAGTTCCTTTAAAACATGCTTTAGATTGGCCAGTTTTTGCTTCTTATGATGAATTAGCAGGTTGTGCTGCATATATGGGTGGTAGGATTCCTACATTTGAAGAAACTAGGTCTATTTATGCCTACGCTGATGCTTTAAAAAAAAAAAAAGAAGCAGAAAGGCAATTAGGTAGGACAGTTCCAGCAGTTAATGCACATTTAACTAATAATGGTGTTGAAATTACACCTCCTTCTTCACCTTCTTCAGAAACACCAGCAGAATCTTCATCACCATCTGATTCTAATACTACTTTGATTACTACAGAAGATTTGTTTTCAGATTTAGATGGTGCAAATGTTGGTTTTCATAATTGGCATCCTATGCCTATTACTTCTAAAGGTAATACTTTAGTTGGTCAAGGTGAATTGGGTGGTGTTTGGGAATGGACTTCTTCAGTTTTGAGGAAATGGGAAGGTTTTGAACCTATGGAATTGTATCCTGGTTATACAGCAGATTTTTTTGATGAAAAACATAATATTGTTTTAGGTGGTTCTTGGGCAACTCATCCTAGGATTGCAGGTAGGAAATCTTTTGTTAATTGGTATCAAAGGAATTATCCTTATGCATGGGTTGGTGCTAGAGTTGTTAGGGATTTATGAGCGCAATGATTGAATAGTCAAAGATTTTTTTTTTTTAATTTTTTTTTTTTAATTTTTTTTTTTTTTCATAGAACTTTTTATTTAAATAAATCACGTCTATATATGTATCAGTATAACGTAAAAAAAAAAACACCGTCAGTTAAACAAAACATAAATAAAAAAAAAAAGAAGTGTCAAATCAAGTGTCAAATCCGCCATCTCTACTCACTCCATAATATTACATATAGATATAGGACAAGCCCGCATTTTCATACTGAAAGGTAAACTTCTATTATTATAGTGGTATCCAACGTTCACCGCTTCCAGCATAGCAGAAATTACGTGTTTTTGCATATGTTATGCTGATCATTGTATGCTTACTACCATTTTTCTTTGCTTCGCCTTGCCTTCTTTGACGTTTTTTTGAAGCAAAAAAAAAGTCAAGACAGATGTGCTTACAAAACCATGTAAGGCTCATTTTCAAAGAAGCTACTAATAGAAAGAGAACAAAGCGTTTACGAGTCTGGAAAATCAATGCCATCTCATTTCGATACTGTTCAACTACACGCCGGCCAAGAGAACCCTGGTGACAATGCTCACAGATCCAGAGCTGTACCAATTTACGCCACCACTTCTTATGTTTTCGAAAACTCTAAGCATGGTTCGCAATTGTTTGGTCTAGAAGTTCCAGGTTACGTCTATTCCCGTTTCCAAAACCCAACCAGTAATGTTTTGGAAGAAAGAATTGCTGCTTTAGAAGGTGGTGCTGCTGCTTTGGCTGTTTCCTCCGGTCAAGCCGCTCAAACCCTTGCCATCCAAGGTTTGGCACACACTGGTGACAACATCGTTTCCACTTCTTACTTATACGGTGGTACTTATAACCAGTTCAAAATCTCGTTCAAAAGATTTGGTATCGAGGCTAGATTTGTTGAAGGTGACAATCCAGAAGAATTCGAAAAGGTCTTTGATGAAAGAACCAAGGCTGTTTATTTGGAAACCATTGGTAATCCAAAGTACAATGTTCCGGATTTTGAAAAAATTGTTGCAATTGCTCACAAACACGGTATTCCAGTTGTCGTTGACAACACATTTGGTGCCGGTGGTTACTTCTGTCAGCCAATTAAATACGGTGCTGATATTGTAACACATTCTGCTACCAAATGGATTGGTGGTCATGGTACTACTATCGGTGGTATTATTGTTGACTCTGGTAAGTTCCCATGGAAGGACTACCCAGAAAAGTTCCCTCAATTCTCTCAACCTGCCGAAGGATATCACGGTACTATCTACAATGAAGCCTACGGTAACTTGGCATACATCGTTCATGTTAGAACTGAACTATTAAGAGATTTGGGTCCATTGATGAACCCATTTGCCTCTTTCTTGCTACTACAAGGTGTTGAAACATTATCTTTGAGAGCTGAAAGACACGGTGAAAATGCATTGAAGTTAGCCAAATGGTTAGAACAATCCCCATACGTATCTTGGGTTTCATACCCTGGTTTAGCATCTCATTCTCATCATGAAAATGCTAAGAAGTATCTATCTAACGGTTTCGGTGGTGTCTTATCTTTCGGTGTAAAAGACTTACCAAATGCCGACAAGGAAACTGACCCATTCAAACTTTCTGGTGCTCAAGTTGTTGACAATTTAAAGCTTGCCTCTAACTTGGCCAATGTTGGTGATGCCAAGACCTTAGTCATTGCTCCATACTTCACTACCCACAAACAATTAAATGACAAAGAAAAGTTGGCATCTGGTGTTACCAAGGACTTAATTCGTGTCTCTGTTGGTATCGAATTTATTGATGACATTATTGCAGACTTCCAGCAATCTTTTGAAACTGTTTTCGCTGGCCAAAAACCATGAATTATACCTCAAGCACGCAGAGAAACCTCTCTTTGGAAAAAAAACATCCAATGAAAAGGCCAGCAATTTCAAGTTAACTCCAAAGAGTATCACTCACTACCAAACAGAATGTTTGAGAAGGAAATGACGCTCAAACAGGCATGCCCCCTGGAATACCAAGGGGCGCAATGTGCGTTCAAAGATTCGATGATTCACGGAATTCTGCAATTCACATTACGTATCGCATTTCGCTGCGTTCTTCATCGATGCGAGAACCAAGAGATCCGTTGTTGAAAGTTTTTAATATTTTAAAATTTCCAGTTACGAAAATTCTTGTTTTTGACAAAAATTTAATGAATAGATAAAATTGTTTGTGTTTGTTACCTCTGGGCC |
| donor DNA-5  (multi-copy integration at δ site for COR biosynthesis, with inducible expression of *CNS1* and *CNS2*) | TTGTTGGAATAAAAATCAACTATCATCTACTAACTAGTATTTACGTTACTAGTATATTATCATATACGGTGTTAGAAGATGACGCAAATGATGAGAAATAGTCATCTAAATTAGTGGAAGCTGAAACGCAAGGATTGATAATGTAATAGGATCAATGAATATTAACATATAAAATGATGATAATAATATTTATAGAATTGTGTAGAATTGCAGATTCCCTTTTATTTTCAACATCGTATTTTCCGAAGCGTTGCATAATGAAAATGTGATAATAAGGCAGTGAGCTACTTTGTCACAACAAAGTACGAGAACAGATGAGGTTCCGTGGTGTTTTTGGGGCAACCGCACTATTTGGAGCGCTGTATATATTTATATCCTACTACAAAAAATCATAAATGACACTGAATTGGACACATGCTAGCGTCAAATACCTTGCCTGGTAAAGTTGTGTGCTAGTGTCTCCCGTCTTCTGTCTATTAGGCGATACCAACCTTGGAACCGAAAACACCAACCTGTCTAGCGTAGTCAGAGAAACCAGTAGCCTTCTCAGCGAAAGCAGATCTAGTACCCATACAGATAGCCTCGATCAGTTCCTCGGCGAAGTATTGGAAAGAAATCTGCCATGGGGAACCTTCTCTAACTCTCTGCTCCCAGTGAACCTTCAATGGCAACCACTCTCTACCTGGCAATCTTTGCTCCAACAAGACTGGGGAACCCTTTCTCTGGATGAATCTCAACTCGTGCTCGTTGTCCAATCTCAGAGTAACGGAGAACAACTCAGTGTCAGCAGCACCATCACAATCAACGGAGTACTTGGTGGACAAACCACAACAACCGTTCATCTGGAAGGCAGATTCAGCGTCTGGAGTCTTAGTAGCACCAGCATCTTCAGTAGGCAATGGACCAATGGAGTTGGAAGTGGACATGTCTTGAACGGAAGCTGGAGTCAAAGAGGATGAAGTCAGCCATTCGATCAGGTCCAAGAAGTGAGTAGCCATAGCCAACATCATACCACCACCAGCAGCCTTAGAAAAACCGGCAGACCAGTGAGTCAACTTGGACAATCTTCTGGTCAGGTACTGGATCTCGATAGCCAAGATGTTGGAAGGCTCTTTGGAAGCAGCCTTCAATTGGATCAGTGGTGGGTAGAATCTCAGTGGATGGTTCATCAATCTCAGCTGGGAAGAACCTTGAGACTGGTTAGCGAAGTGTCTCATAGTAGCCATGTCCAAAGCCAATGGCTTCTCGCAAACGATGTCGAAACCACCAAGATCCAAAGCCTCTTGAACCAAAGCTGCGTGCTTGTCATGTGGGGAAGCAACGAACAACAAGTTAGCACCGTGAGTAGCTTGAACCTCAGCCAGGGAAACGTCAATACATGGAATACCGTGCTTGTTACCAACGGCCTCAGTCTTAGCTCTGTCTCTTCCACCACACAAACCAACAACCTCAACACCTCTTCTCTGCAAGGCTGGCAAAATAGTCTTGACACCGTAACCAGTACCGACGATGATAGCCTTGGGTCTTTCGATAACACCGAAAGCCTCCAAAACCAAGTGAGCGGATCTAGCCAAGAACAAGTCCTTGTTTCTGTGGACAGTCTCGTTTCTGGAGGTCAAGTCGAATGGAACGGTCATACCCAATTCCTCTGGCCTTCTCAAGTAGTCGGTGATGAACATCAATGGCAGCAACTGGACTCTTTCCATGGATGGGTTTTCTTCGTGAACGTCAGTCAAGGCTCTAGCCATTTGAGCCAATTCGTTGTCAACGGAAGAGGCACCATGAGCTTCAACTTGAGTTCTGAAGTCGACGTCCTGTTCAACGATAGTTGGAGTGGAAACGTGCAGTCCTCTGTCGAAAGTTGGAAATCTGGAGGACAGTGGGTTGATTGGCAAGGCAGAATGGTCATCACCATGCCAACAAGCTTGACCCTTATCGAACACGCAGTATCTGGTTGGGGAGTAGATAGTTCTGTGGATATCACCAGGGGACAGCAAAGTACCCTGCTTAGCAATGTAACAGATGGCTCTTGGCCTTCTGGCAGCCAATTCTCTAACAACTCTACCACCAAGGTTACCCCAGTAGGAGAACTTAGCACCAATCAAGGAGAAAACCAGACCGGGCCTCAATTCCAATCTAACCAAACCGTAGTACTGAGCGTCCTGTCTACCAATAACTCTGGCAGTTGGAACCAAAGAGGCGAATCTTGGCAACAGCTCAGCAACAAAACCCAAGATCAGGATGTCACCTGGCTCAACAGACAAAGAGAACTCGGTCAAACCAGTCCAAGCCATCATAGACTGAGTCAAGTGTGGGTACAACACGCATCTGATTGGACCGTGATGAGAAGAAATGGCACCGTCGTGTTGAGCAACCTCCAAAATGGAAGCGTAGTGTCTGATGTAGTCCTCACCTGGCATAATGGTCAACAGGGCAACGATTCTTTGAGAACCAGACTCTTGGGCCAACAGCAACTCTGGTCTAAGAAAGTTGTATGGCTTGTGCAGGTCGTGGGTTCTCCAAACGAATCTTTCAACCCAGTCGAAACCGAAAGCACCGTGGTGTTTTTGCAAGTACTGGAATGGAGCAGCGTGCAATTCAACTGGGATCTTCAACTGACCCAATCTCTCCAAGTGCTGCATTGGCAATCTGGACAAGCAGTACTCGATCAAGGCGTAGGACATAGTGTGGTTAGCAACAGTTGGCAATGGGAAGTCAACCAACTGTCTCAAGGCAACACCGTTAGACCAAGTTCTTCTGAAAGCTGGGTCGAATGGTTGTCTCAGAGCATCTCTGTGGTTTTCCCTTTCGAAGGATGGACAGGTAGTTGGGTAACCGTTTTCGTTCATGGCCATTTTCAAAAATTCTTACTTTTTTTTTGGATGGACGCAAAGAAGTTTAATAATCATATTACATGGCATTACCACCATATACATATCCATATACATATCCATATCTAATCTTACTTATATGTTGTGGAAATGTAAAGAGCCCCATTATCTTAGCCTAAAAAAACCTTCTCTTTGGAACTTTCAGTAATACGCTTAACTGCTCATTGCTATATTGAAGTACGGATTAGAAGCCGCCGAGCGGGTGACAGCCCTCCGAAGGAAGACTCTCCTCCGTGCGTCCTCGTCTTCACCGGTCGCGTTCCTGAAACGCAGATGTGCCTCGCGCCGCACTGCTCCGAACAATAAAGATTCTACAATACTAGCTTTTATGGTTATGAAGAGGAAAAATTGGCAGTAACCTGGCCCCACAAACCTTCAAATGAACGAATCAAATTAACAACCATAGGATGATAATGCGATTAGTTTTTTAGCCTTATTTCTGGGGTAATTAATCAGCGAAGCGATGATTTTTGATCTATTAACAGATATATAAATGCAAAAACTGCATAACCACTTTAACTAATACTTTCAACATTTTCGGTTTGTATTACTTCTTATTCAAATGTAATAAAAGTATCAACAAAAAATTGTTAATATACCTCTATACTTTAACGTCAAGGAGAAAAAACATGTCCTGTCCAACTTCTGCTGGTGTCTTGCAAACTCACCAGTTGTTGAACGACAACTCCATCTTGATCAGGGACGAGATCTACGGTGAAGAGTTGGTTTCTGAGCCAGTCTTGGTTGAGTTGTTGCAATCCGCTGAGGTTCAGAGATTGCAGGGTATTTGTCAGCACGGTGTTACCGGTTTCTTGGGTATCACTCCAAGAGTCACTAGATTGGAGCATTCCGTTGGTGCCTTCATCCTGGTTAGAAGAGTCGGTGCTGCTTTGGACGAACAAGTTGCAGCTTTGTTGCACGACATTTCCCACACTACTTTGTCCCACGTTATCGACCACGCTTTGTCTAAGCCTGGTGAAGGTTCTTACCACGAGGTTCACAAGGCCAGATACCTTAAGACTACCAGATTGCCAGACATCGTTGCCAAGCACGGTATTTCCCAAAAGGTGTTCGAGGAAGAGTTGTTCCCATTGGTCGAAATGCCATCTCCACAGTTGTGTGCTGACAGATTGGACTACGCTTTGAGAGATGCTGTCTCCTTCGGTAAGTTGGCTATGGAAGATGCTCAAAAGGTCGTGTCCTCCTTGAGAGCTTTTCCATCTGCTACTACTGCCAGAAGATTGCTGGTTTTGGACGACGCTGAAGTTGCTTTGACTTTGTCCAGAGCTTACACCACTACCGACAAGGACGTTTGGTCTAACCCAGCTCACATTGACATGTACGAGAGAACCGGTAGAGTCATCGGTGAATTGGTTGAAGCTGGTTCCGTTGAGGACAAGGTTTTGTGGCAAGTTTCTGACGCTGAGTTCTGGACCATGTTGAGACAAGCTGCTAACCCAGAACAGAGAAGGGCTATTGAGAGATTGGAGACTGAAGGTGTTCCAGAGGATGACGGTTTGGAATTGCCACACTGTGCTAAGATCAGAACCTTGGATCCAGACGTTTGGCAGAGAGGTGAAAAGCAACCAGCTCCATTGTCCATCGTTTTGCCAACTTGGGGTACTGAGAGACAGCAGTACATCTTGTCCAGGACTCAACACAGATAGTAAGCGCAATGATTGAATAGTCAAAGATTTTTTTTTTTTAATTTTTTTTTTTTAATTTTTTTTTTTTTTCATAGAACTTTTTATTTAAATAAATCACGTCTATATATGTATCAGTATAACGTAAAAAAAAAAACACCGTCAGTTAAACAAAACATAAATAAAAAAAAAAAGAAGTGTCAAATCAAGTGTCAAATCCGCCATCTCTACTCACTCCATAATATTACATATAGATATAGGACAAGCCCGCATTTTCATACTGAAAGGTAAACTTCTATTATTATAGTGGTATCCAACGTTCACCGCTTCCAGCATAGCAGAAATTACGTGTTTTTGCATATGTTATGCTGATCATTGTATGCTTACTACCATTTTTCTTTGCTTCGCCTTGCCTTCTTTGACGTTTTTTTGAAGCAAAAAAAAAGTCAAGACAGATGTGCTTACAAAACCATGTAAGGCTCATTTTCAAAGAAGCTACTAATAGAAAGAGAACAAAGCGTTTACGAGTCTGGAAAATCAATGCCATCTCATTTCGATACTGTTCAACTACACGCCGGCCAAGAGAACCCTGGTGACAATGCTCACAGATCCAGAGCTGTACCAATTTACGCCACCACTTCTTATGTTTTCGAAAACTCTAAGCATGGTTCGCAATTGTTTGGTCTAGAAGTTCCAGGTTACGTCTATTCCCGTTTCCAAAACCCAACCAGTAATGTTTTGGAAGAAAGAATTGCTGCTTTAGAAGGTGGTGCTGCTGCTTTGGCTGTTTCCTCCGGTCAAGCCGCTCAAACCCTTGCCATCCAAGGTTTGGCACACACTGGTGACAACATCGTTTCCACTTCTTACTTATACGGTGGTACTTATAACCAGTTCAAAATCTCGTTCAAAAGATTTGGTATCGAGGCTAGATTTGTTGAAGGTGACAATCCAGAAGAATTCGAAAAGGTCTTTGATGAAAGAACCAAGGCTGTTTATTTGGAAACCATTGGTAATCCAAAGTACAATGTTCCGGATTTTGAAAAAATTGTTGCAATTGCTCACAAACACGGTATTCCAGTTGTCGTTGACAACACATTTGGTGCCGGTGGTTACTTCTGTCAGCCAATTAAATACGGTGCTGATATTGTAACACATTCTGCTACCAAATGGATTGGTGGTCATGGTACTACTATCGGTGGTATTATTGTTGACTCTGGTAAGTTCCCATGGAAGGACTACCCAGAAAAGTTCCCTCAATTCTCTCAACCTGCCGAAGGATATCACGGTACTATCTACAATGAAGCCTACGGTAACTTGGCATACATCGTTCATGTTAGAACTGAACTATTAAGAGATTTGGGTCCATTGATGAACCCATTTGCCTCTTTCTTGCTACTACAAGGTGTTGAAACATTATCTTTGAGAGCTGAAAGACACGGTGAAAATGCATTGAAGTTAGCCAAATGGTTAGAACAATCCCCATACGTATCTTGGGTTTCATACCCTGGTTTAGCATCTCATTCTCATCATGAAAATGCTAAGAAGTATCTATCTAACGGTTTCGGTGGTGTCTTATCTTTCGGTGTAAAAGACTTACCAAATGCCGACAAGGAAACTGACCCATTCAAACTTTCTGGTGCTCAAGTTGTTGACAATTTAAAGCTTGCCTCTAACTTGGCCAATGTTGGTGATGCCAAGACCTTAGTCATTGCTCCATACTTCACTACCCACAAACAATTAAATGACAAAGAAAAGTTGGCATCTGGTGTTACCAAGGACTTAATTCGTGTCTCTGTTGGTATCGAATTTATTGATGACATTATTGCAGACTTCCAGCAATCTTTTGAAACTGTTTTCGCTGGCCAAAAACCATGAGGCTGGCAACTAATAGGGACACTACCAATATATTATCATATACGGTGTTAGACGATGACATAAGATACGAGGAACTGTCATCGAAGTTAGAGGAAGCTGAAATGCAAGGATTGATAATGTAATAGGATAATGAAACATATAAAACGGAATGAGGAATAATCGTAATATTAGTATATAGAGATAAAGATTCCATTTTGAGGATTCCTATATCCTCGAGGAGAACTTCTAGTATATTCTGTATACCTGATATTATAGCCT |
| donor DNA-6  (multi-copy integration at rDNA site for COR biosynthesis, with inducible expression of *CNS1* and *CNS2*) | TCTATGACGTCCTGTTCCAAGGAACATAGACAAGGAACGGCCCCAAAGTTGCCCTCTCCAAATTACAACTCGGGCACCGAAGGTACCAGATTTCAAATTTGAGCTTTTGCCGCTTCACTCGCCGTTACTAAGGCAATCCCGGTTGGTTTCTTTTCCTCCGCTTATTGATATGCTTAAGTTCAGCGGGTACTCCTACCTGATTTGAGGTCAAACTTTAAGAACATTGTTCGCCTAGACGCTCTCTTCTTATCGATAACGTTCCAATACGCTCAGTATAAAAAAAGATTAGCCGCAGTTGGTAAAACCTAAAACGACCGTACTTGCATTTTCAACATCGTATTTTCCGAAGCGTTGCATAATGAAAATGTGATAATAAGGCAGTGAGCTACTTTGTCACAACAAAGTACGAGAACAGATGAGGTTCCGTGGTGTTTTTGGGGCAACCGCACTATTTGGAGCGCTGTATATATTTATATCCTACTACAAAAAATCATAAATGACACTGAATTGGACACATGCTAGCGTCAAATACCTTGCCTGGTAAAGTTGTGTGCTAGTGTCTCCCGTCTTCTGTCTATTAGGCGATACCAACCTTGGAACCGAAAACACCAACCTGTCTAGCGTAGTCAGAGAAACCAGTAGCCTTCTCAGCGAAAGCAGATCTAGTACCCATACAGATAGCCTCGATCAGTTCCTCGGCGAAGTATTGGAAAGAAATCTGCCATGGGGAACCTTCTCTAACTCTCTGCTCCCAGTGAACCTTCAATGGCAACCACTCTCTACCTGGCAATCTTTGCTCCAACAAGACTGGGGAACCCTTTCTCTGGATGAATCTCAACTCGTGCTCGTTGTCCAATCTCAGAGTAACGGAGAACAACTCAGTGTCAGCAGCACCATCACAATCAACGGAGTACTTGGTGGACAAACCACAACAACCGTTCATCTGGAAGGCAGATTCAGCGTCTGGAGTCTTAGTAGCACCAGCATCTTCAGTAGGCAATGGACCAATGGAGTTGGAAGTGGACATGTCTTGAACGGAAGCTGGAGTCAAAGAGGATGAAGTCAGCCATTCGATCAGGTCCAAGAAGTGAGTAGCCATAGCCAACATCATACCACCACCAGCAGCCTTAGAAAAACCGGCAGACCAGTGAGTCAACTTGGACAATCTTCTGGTCAGGTACTGGATCTCGATAGCCAAGATGTTGGAAGGCTCTTTGGAAGCAGCCTTCAATTGGATCAGTGGTGGGTAGAATCTCAGTGGATGGTTCATCAATCTCAGCTGGGAAGAACCTTGAGACTGGTTAGCGAAGTGTCTCATAGTAGCCATGTCCAAAGCCAATGGCTTCTCGCAAACGATGTCGAAACCACCAAGATCCAAAGCCTCTTGAACCAAAGCTGCGTGCTTGTCATGTGGGGAAGCAACGAACAACAAGTTAGCACCGTGAGTAGCTTGAACCTCAGCCAGGGAAACGTCAATACATGGAATACCGTGCTTGTTACCAACGGCCTCAGTCTTAGCTCTGTCTCTTCCACCACACAAACCAACAACCTCAACACCTCTTCTCTGCAAGGCTGGCAAAATAGTCTTGACACCGTAACCAGTACCGACGATGATAGCCTTGGGTCTTTCGATAACACCGAAAGCCTCCAAAACCAAGTGAGCGGATCTAGCCAAGAACAAGTCCTTGTTTCTGTGGACAGTCTCGTTTCTGGAGGTCAAGTCGAATGGAACGGTCATACCCAATTCCTCTGGCCTTCTCAAGTAGTCGGTGATGAACATCAATGGCAGCAACTGGACTCTTTCCATGGATGGGTTTTCTTCGTGAACGTCAGTCAAGGCTCTAGCCATTTGAGCCAATTCGTTGTCAACGGAAGAGGCACCATGAGCTTCAACTTGAGTTCTGAAGTCGACGTCCTGTTCAACGATAGTTGGAGTGGAAACGTGCAGTCCTCTGTCGAAAGTTGGAAATCTGGAGGACAGTGGGTTGATTGGCAAGGCAGAATGGTCATCACCATGCCAACAAGCTTGACCCTTATCGAACACGCAGTATCTGGTTGGGGAGTAGATAGTTCTGTGGATATCACCAGGGGACAGCAAAGTACCCTGCTTAGCAATGTAACAGATGGCTCTTGGCCTTCTGGCAGCCAATTCTCTAACAACTCTACCACCAAGGTTACCCCAGTAGGAGAACTTAGCACCAATCAAGGAGAAAACCAGACCGGGCCTCAATTCCAATCTAACCAAACCGTAGTACTGAGCGTCCTGTCTACCAATAACTCTGGCAGTTGGAACCAAAGAGGCGAATCTTGGCAACAGCTCAGCAACAAAACCCAAGATCAGGATGTCACCTGGCTCAACAGACAAAGAGAACTCGGTCAAACCAGTCCAAGCCATCATAGACTGAGTCAAGTGTGGGTACAACACGCATCTGATTGGACCGTGATGAGAAGAAATGGCACCGTCGTGTTGAGCAACCTCCAAAATGGAAGCGTAGTGTCTGATGTAGTCCTCACCTGGCATAATGGTCAACAGGGCAACGATTCTTTGAGAACCAGACTCTTGGGCCAACAGCAACTCTGGTCTAAGAAAGTTGTATGGCTTGTGCAGGTCGTGGGTTCTCCAAACGAATCTTTCAACCCAGTCGAAACCGAAAGCACCGTGGTGTTTTTGCAAGTACTGGAATGGAGCAGCGTGCAATTCAACTGGGATCTTCAACTGACCCAATCTCTCCAAGTGCTGCATTGGCAATCTGGACAAGCAGTACTCGATCAAGGCGTAGGACATAGTGTGGTTAGCAACAGTTGGCAATGGGAAGTCAACCAACTGTCTCAAGGCAACACCGTTAGACCAAGTTCTTCTGAAAGCTGGGTCGAATGGTTGTCTCAGAGCATCTCTGTGGTTTTCCCTTTCGAAGGATGGACAGGTAGTTGGGTAACCGTTTTCGTTCATGGCCATTTTCAAAAATTCTTACTTTTTTTTTGGATGGACGCAAAGAAGTTTAATAATCATATTACATGGCATTACCACCATATACATATCCATATACATATCCATATCTAATCTTACTTATATGTTGTGGAAATGTAAAGAGCCCCATTATCTTAGCCTAAAAAAACCTTCTCTTTGGAACTTTCAGTAATACGCTTAACTGCTCATTGCTATATTGAAGTACGGATTAGAAGCCGCCGAGCGGGTGACAGCCCTCCGAAGGAAGACTCTCCTCCGTGCGTCCTCGTCTTCACCGGTCGCGTTCCTGAAACGCAGATGTGCCTCGCGCCGCACTGCTCCGAACAATAAAGATTCTACAATACTAGCTTTTATGGTTATGAAGAGGAAAAATTGGCAGTAACCTGGCCCCACAAACCTTCAAATGAACGAATCAAATTAACAACCATAGGATGATAATGCGATTAGTTTTTTAGCCTTATTTCTGGGGTAATTAATCAGCGAAGCGATGATTTTTGATCTATTAACAGATATATAAATGCAAAAACTGCATAACCACTTTAACTAATACTTTCAACATTTTCGGTTTGTATTACTTCTTATTCAAATGTAATAAAAGTATCAACAAAAAATTGTTAATATACCTCTATACTTTAACGTCAAGGAGAAAAAACATGTCCTGTCCAACTTCTGCTGGTGTCTTGCAAACTCACCAGTTGTTGAACGACAACTCCATCTTGATCAGGGACGAGATCTACGGTGAAGAGTTGGTTTCTGAGCCAGTCTTGGTTGAGTTGTTGCAATCCGCTGAGGTTCAGAGATTGCAGGGTATTTGTCAGCACGGTGTTACCGGTTTCTTGGGTATCACTCCAAGAGTCACTAGATTGGAGCATTCCGTTGGTGCCTTCATCCTGGTTAGAAGAGTCGGTGCTGCTTTGGACGAACAAGTTGCAGCTTTGTTGCACGACATTTCCCACACTACTTTGTCCCACGTTATCGACCACGCTTTGTCTAAGCCTGGTGAAGGTTCTTACCACGAGGTTCACAAGGCCAGATACCTTAAGACTACCAGATTGCCAGACATCGTTGCCAAGCACGGTATTTCCCAAAAGGTGTTCGAGGAAGAGTTGTTCCCATTGGTCGAAATGCCATCTCCACAGTTGTGTGCTGACAGATTGGACTACGCTTTGAGAGATGCTGTCTCCTTCGGTAAGTTGGCTATGGAAGATGCTCAAAAGGTCGTGTCCTCCTTGAGAGCTTTTCCATCTGCTACTACTGCCAGAAGATTGCTGGTTTTGGACGACGCTGAAGTTGCTTTGACTTTGTCCAGAGCTTACACCACTACCGACAAGGACGTTTGGTCTAACCCAGCTCACATTGACATGTACGAGAGAACCGGTAGAGTCATCGGTGAATTGGTTGAAGCTGGTTCCGTTGAGGACAAGGTTTTGTGGCAAGTTTCTGACGCTGAGTTCTGGACCATGTTGAGACAAGCTGCTAACCCAGAACAGAGAAGGGCTATTGAGAGATTGGAGACTGAAGGTGTTCCAGAGGATGACGGTTTGGAATTGCCACACTGTGCTAAGATCAGAACCTTGGATCCAGACGTTTGGCAGAGAGGTGAAAAGCAACCAGCTCCATTGTCCATCGTTTTGCCAACTTGGGGTACTGAGAGACAGCAGTACATCTTGTCCAGGACTCAACACAGATAGTAAGCGCAATGATTGAATAGTCAAAGATTTTTTTTTTTTAATTTTTTTTTTTTAATTTTTTTTTTTTTTCATAGAACTTTTTATTTAAATAAATCACGTCTATATATGTATCAGTATAACGTAAAAAAAAAAACACCGTCAGTTAAACAAAACATAAATAAAAAAAAAAAGAAGTGTCAAATCAAGTGTCAAATCCGCCATCTCTACTCACTCCATAATATTACATATAGATATAGGACAAGCCCGCATTTTCATACTGAAAGGTAAACTTCTATTATTATAGTGGTATCCAACGTTCACCGCTTCCAGCATAGCAGAAATTACGTGTTTTTGCATATGTTATGCTGATCATTGTATGCTTACTACCATTTTTCTTTGCTTCGCCTTGCCTTCTTTGACGTTTTTTTGAAGCAAAAAAAAAGTCAAGACAGATGTGCTTACAAAACCATGTAAGGCTCATTTTCAAAGAAGCTACTAATAGAAAGAGAACAAAGCGTTTACGAGTCTGGAAAATCAATGCCATCTCATTTCGATACTGTTCAACTACACGCCGGCCAAGAGAACCCTGGTGACAATGCTCACAGATCCAGAGCTGTACCAATTTACGCCACCACTTCTTATGTTTTCGAAAACTCTAAGCATGGTTCGCAATTGTTTGGTCTAGAAGTTCCAGGTTACGTCTATTCCCGTTTCCAAAACCCAACCAGTAATGTTTTGGAAGAAAGAATTGCTGCTTTAGAAGGTGGTGCTGCTGCTTTGGCTGTTTCCTCCGGTCAAGCCGCTCAAACCCTTGCCATCCAAGGTTTGGCACACACTGGTGACAACATCGTTTCCACTTCTTACTTATACGGTGGTACTTATAACCAGTTCAAAATCTCGTTCAAAAGATTTGGTATCGAGGCTAGATTTGTTGAAGGTGACAATCCAGAAGAATTCGAAAAGGTCTTTGATGAAAGAACCAAGGCTGTTTATTTGGAAACCATTGGTAATCCAAAGTACAATGTTCCGGATTTTGAAAAAATTGTTGCAATTGCTCACAAACACGGTATTCCAGTTGTCGTTGACAACACATTTGGTGCCGGTGGTTACTTCTGTCAGCCAATTAAATACGGTGCTGATATTGTAACACATTCTGCTACCAAATGGATTGGTGGTCATGGTACTACTATCGGTGGTATTATTGTTGACTCTGGTAAGTTCCCATGGAAGGACTACCCAGAAAAGTTCCCTCAATTCTCTCAACCTGCCGAAGGATATCACGGTACTATCTACAATGAAGCCTACGGTAACTTGGCATACATCGTTCATGTTAGAACTGAACTATTAAGAGATTTGGGTCCATTGATGAACCCATTTGCCTCTTTCTTGCTACTACAAGGTGTTGAAACATTATCTTTGAGAGCTGAAAGACACGGTGAAAATGCATTGAAGTTAGCCAAATGGTTAGAACAATCCCCATACGTATCTTGGGTTTCATACCCTGGTTTAGCATCTCATTCTCATCATGAAAATGCTAAGAAGTATCTATCTAACGGTTTCGGTGGTGTCTTATCTTTCGGTGTAAAAGACTTACCAAATGCCGACAAGGAAACTGACCCATTCAAACTTTCTGGTGCTCAAGTTGTTGACAATTTAAAGCTTGCCTCTAACTTGGCCAATGTTGGTGATGCCAAGACCTTAGTCATTGCTCCATACTTCACTACCCACAAACAATTAAATGACAAAGAAAAGTTGGCATCTGGTGTTACCAAGGACTTAATTCGTGTCTCTGTTGGTATCGAATTTATTGATGACATTATTGCAGACTTCCAGCAATCTTTTGAAACTGTTTTCGCTGGCCAAAAACCATGAATTATACCTCAAGCACGCAGAGAAACCTCTCTTTGGAAAAAAAACATCCAATGAAAAGGCCAGCAATTTCAAGTTAACTCCAAAGAGTATCACTCACTACCAAACAGAATGTTTGAGAAGGAAATGACGCTCAAACAGGCATGCCCCCTGGAATACCAAGGGGCGCAATGTGCGTTCAAAGATTCGATGATTCACGGAATTCTGCAATTCACATTACGTATCGCATTTCGCTGCGTTCTTCATCGATGCGAGAACCAAGAGATCCGTTGTTGAAAGTTTTTAATATTTTAAAATTTCCAGTTACGAAAATTCTTGTTTTTGACAAAAATTTAATGAATAGATAAAATTGTTTGTGTTTGTTACCTCTGGGCC |

**Tab. S6 Composition of the synthetic complete (SC) medium used in this study.**

| **Component** | **Level (per liter)** |
| --- | --- |
| Glucose/Galactose | 30 g |
| Ammonium sulfate | 5.0 g |
| L-Arginine (Arg) | 0.076 g |
| L-Cysteine (Cys) | 0.076 g |
| L-Lysine (Lys) | 0.076 g |
| L-Threonine (Thr) | 0.076 g |
| L-Aspartic acid (Asp) | 0.076 g |
| L-Isoleucine (Ile) | 0.076 g |
| L-Phenylalanine (Phe) | 0.076 g |
| L-Proline (Pro) | 0.076 g |
| L-Serine (Ser) | 0.076 g |
| L-Tyrosine (Tyr) | 0.076 g |
| L-Valine (Val) | 0.076 g |
| L-Tryptophan (Trp) | 0.076 g |
| L-Glutamic acid (Glu) | 0.076 g |
| L-Glutamine (Gln) | 0.076 g |
| L-Glycine (Gly) | 0.076 g |
| L-Alanine (Ala) | 0.076 g |
| L-Asparagine (Asn) | 0.076 g |
| Inositol | 0.076 g |
| p-Aminobenzoic acid | 0.008 g |
| Biotin | 2 μg |
| Pantothenic acid (Calcium Pantothenate) | 400 μg |
| Folic acid | 2 μg |
| Inositol | 2 mg |
| Niacin (Nicotinic acid) | 400 μg |
| p-Aminobenzoic acid | 200 μg |
| Pyridoxine HCl (Vitamin B6) | 400 μg |
| Riboflavin (Vitamin B2) | 200 μg |
| Thiamine HCl (Vitamin B1) | 400 μg |
| Boric acid | 500 μg |
| Copper sulfate | 40 μg |
| Potassium iodide | 100 μg |
| Ferric chloride | 200 μg |
| Manganese sulfate | 400 μg |
| Sodium molybdate | 200 μg |
| Zinc sulfate | 400 μg |
| Ferrous sulfate | 0.02 mg |
| Potassium dihydrogen phosphate | 1.0 g |
| Magnesium sulfate | 0.5 g |
| Sodium chloride | 0.1 g |
| Calcium chloride | 0.1 g |
| L-Methionine (Met) | 0.1 g |
| L-Histidine (His) | 0.1 g |
| L-Leucine (Leu) | 0.1 g |
| L-Uracil (Ura) | 0.1 g |


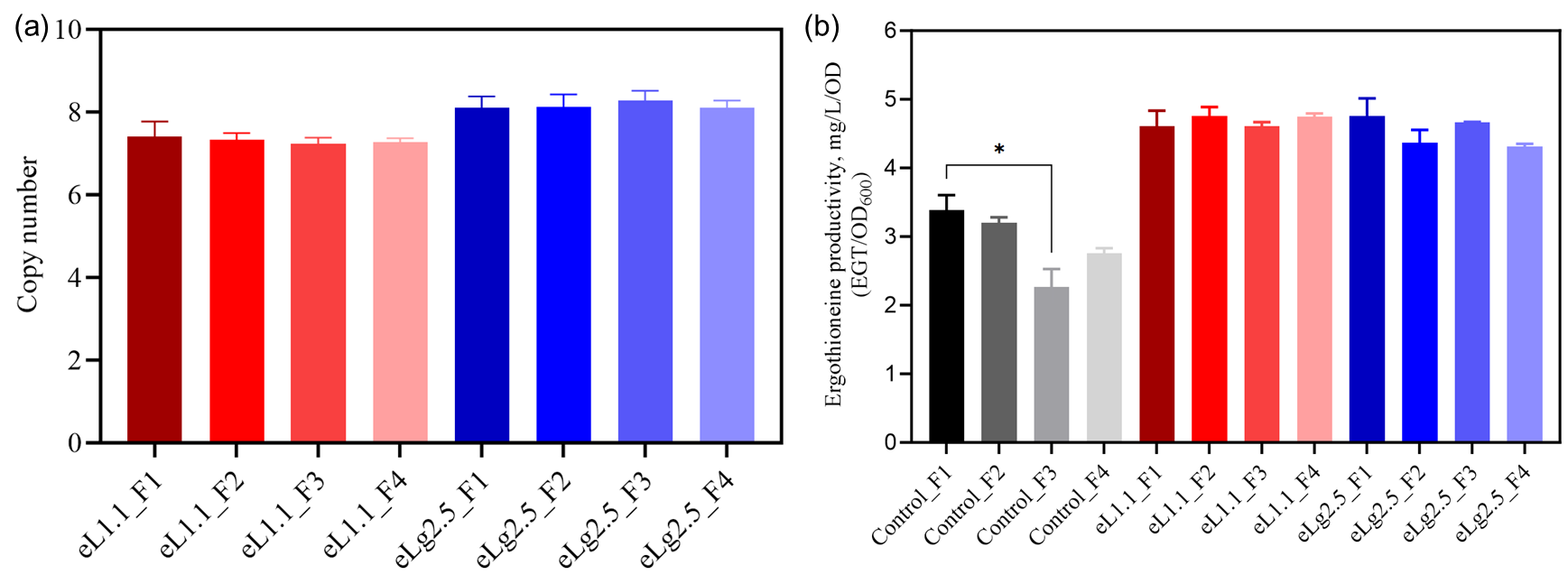


**Fig. S1 Passage stability and fermentation performance analysis.**

(a) Copy number of the 8-copy strains (the constitutive expression strain eL1.1_F1-4, and the inducible expression strain eLg2.5_F1-4); (b) Ergothioneine productivity (mg/L/OD) for the 8-copy strains (the constitutive expression strain eL1.1_F1-4, and the inducible expression strain eLg2.5_F1-4) and the control (episomal expression with plasmid, Control_F1-4). * represents p<0.05.
